# Morpho-anatomical characterization, DNA barcoding, and phylogenetic analysis of *Solanum argentinum* Bitter & Lillo: an addition to the alien Flora of Egypt

**DOI:** 10.1186/s12870-026-10047-1

**Published:** 2026-11-03

**Authors:** Mostafa Aboulela, Rim S. Hamdy, Ahmed K. Osman, Iman H. Nour, Mohamed O. Badry

**Affiliations:** 1 Botany and Microbiology Department, Faculty of Science, Assiut University, Assiut, 71515 Egypt; 2 Department of Botany and Microbiology, Faculty of Science, Cairo University, Giza, 12613 Egypt; 3 Department of Botany and Microbiology, Faculty of Science, Qena University, Qena, 83523 Egypt; 4 Botany and Microbiology Department, Faculty of Science, Alexandria University, Alexandria, 21515 Egypt

**Keywords:** DNA barcoding, Egypt, Geminata clade, Introduced species, ITS2, *rbcL*, *Solanum argentinum*, *trnL*

## Abstract

**Background:**

*Solanum argentinum *Bitter & Lillo, a woody species native to South America (Argentina, Bolivia, and Paraguay), is documented as an addition to the alien flora of Egypt. This study presents the species’ first published record in Egypt, based on specimens collected from the Giza Zoological Garden, where it has persisted for over 60 years.

**Results:**

Morphological and microscopic characterization of the Egyptian material revealed key diagnostic features, including an unarmed woody habit, dendritic non-glandular trichomes, white stellate corollas, and globose yellowish-orange berries (3.5–6 mm in height and diameter) with 4–8 reniform, minutely pitted seeds. Scanning electron microscopy (SEM) showed small, tricolporate, prolate pollen grains (20–25 × 12–16 μm) with scabrate ornamentation. Foliar epidermal analysis showed hypostomatic, anisocytic stomata (rarely anomocytic) on the abaxial surface with an average stomatal density of 186 per mm^2^, and a stomatal index of 26.4%. Anatomical sections of vegetative organs confirmed diagnostic family- and genus-level traits, including bicollateral vascular bundles and sand crystals. Multi-locus phylogenetic analyses of a single Egyptian specimen (voucher ASTU-MA1001), using DNA sequence data from the nuclear internal transcribed spacer (ITS2) and chloroplast (*rbcL* and *trnL)* barcoding markers, placed the Egyptian specimen firmly within the Geminata clade, with close affinity to reference *S. argentinum* sequences. Re-evaluation of CAI and Kew historical sheets (Mohamed El Mahdi s.n., collected in December 1963) confirmed its continuous casual persistence inside the zoo.

**Conclusion:**

This study provides the first published record of *S. argentinum* in Egypt. Because the species remains restricted to a single locality, we classified it as an introduced casual alien. The comprehensive morphological, anatomical, and molecular dataset presented here provides essential diagnostic tools to differentiate this taxon from other native and cultivated *Solanum* species in Egypt, establishing a robust baseline for long-term monitoring to track potential escape or naturalization.

**Supplementary Information:**

The online version contains supplementary material available at https://doi.org/10.1186/s12870-026-10047-1.

## Introduction

The mega-diverse *Solanum* L., with more than 1237 accepted species, is the largest in the family Solanaceae [1]. It is considered among the most species-rich angiosperm genera [2–4]. The genus includes several economically important species (i.e., potato, tomato, and eggplant). It occupies a wide range of habitats and habits, is widely distributed throughout temperate and tropical regions, and exhibits remarkable morphological and ecological diversity, spanning from minute ephemeral herbs and herbaceous climbers to shrubs and large trees [5].

Morphologically, *Solanum* is characterized by a relatively uniform floral structure, including the pentamerous, sympetalous flowers with rotate to star-shaped corollas and a prominent central stamen cone [6–8]. The stamen cone consists of very short filaments and anthers, which exhibit poricidal dehiscence and release pollen through apical pores rather than longitudinal slits [7–9]. Most *Solanum* species produce spherical berries of diverse colors (e.g., red, yellow, orange, or black). These berries contain a few to many compressed, reniform seeds [6, 9–11].

Traditionally, identifying *Solanum* species has relied heavily on morphology; however, high species richness, phenotypic plasticity, and hybridization often make this approach alone challenging [5, 7]. To ensure accurate identification, taxonomists often integrate additional diagnostic tools, such as comparative anatomy, palynology, and molecular data. In addition, micro-morphological features, such as trichome diversity and pollen and seed characteristics, have proven vital diagnostic tools for differentiating species within this taxonomically complex genus [3, 9, 12–14].

The leaf epidermal features of the genus *Solanum* are characterized by significant phenotypic plasticity and various micro-morphological details that serve as vital diagnostic tools for taxonomic and infrageneric delimitation. The indumentum covering the surface of the genus *Solanum* is composed of diverse types of glandular and non-glandular trichomes, providing valuable sources of information for the systematics of the genus *Solanum* [12, 14–18]. For example, the non-glandular trichomes exhibit remarkable structural diversity, including simple, compound, dendritic (tree-like), stellate (star-shaped), peltate, and other forms [12, 14, 16, 18]. This huge diversity of trichome types allowed taxonomists to distinguish different clades of the genus *Solanum*; for example, the Leptostemonum clade is characterized by presence of stellate hairs, whereas Geminata clade members contain simple and dendritic trichomes but never stellate ones [6, 8, 19].

Integrating molecular data, particularly the internal transcribed spacer (ITS2) and various chloroplast markers (*ndhF*, *rbcL*, *trnS-G*, and *trnT-F*), has revolutionized the Solanaceae taxonomy by resolving phylogenetic relationships that gross morphology often fails to clarify [5, 20–31]. These nuclear and chloroplast DNA markers have been instrumental in identifying the major monophyletic groups within *Solanum* and constructing the best-resolved phylogenetic trees to date [23, 24, 29, 32–34]. The molecular phylogenetic studies have classified the genus *Solanum* into 12–15 major monophyletic groups, including the Leptostemonum clade (spiny solanums), the Morelloid clade (black nightshades), the Potato clade, the Pteroidea clade, the Crinitum clade, the Androceras clade, the Holophylla clade, and the Geminata clade [24, 29].

The Geminata clade is the largest monophyletic group of the non-spiny *Solanum* species, and it includes about 157 species to date [35]. Geminata clade members are characterized by the unarmed woody habit, monochasial branching, opposite leaves (rarely alternate), terminal and/or internodal inflorescences, and usually white or greenish-white flowers [6, 8, 19, 35]. The clade is also distinguished by unicellular, multicellular uniseriate, anddendritic trichomes, but never stellate [6, 8]. *Solanum argentinum* Bitter & Lillo belongs to the *Solanum pseudocapsicum* species group of the Geminata clade. It is a perennial, woody shrub native to South America (in Argentina, Bolivia, and Paraguay). The species is distinguished by its elliptic to narrowly elliptic leaves, dendritic trichomes, globose orange or yellowish-orange berries, and a small number of seeds per fruit [6, 8, 36].

*S. argentinum* has been the subject of a few taxonomic studies focusing on the morphological descriptions [6, 36], leaf surface micro-morphology [37], fruit and seed development [38], seed lipid components [39], karyotype analysis [40], and molecular data [24, 32]. However, detailed studies of the micro-morphological, palynological, and seed characteristics of *S. argentinum* are lacking.

Bitter [36] distinguished the species as new and widely distributed throughout central and northwest Argentina, with records from Córdoba, Santiago del Estero, Tucumán, and Catamarca (including Bajada). Also, *S. argentinum* var. *chroniotrichum* Bitter was recognized based on stem and stem branch traits and leaf density, color, and trichome type and length. This variety and *S. chroniotrichum* (Bitter) C.V.Morton are now treated as synonyms of *S. argentinum* based on pubescence density investigated by Knapp [6], who reported that pubescence classes overlap and show no effect across environmental gradients (latitude, longitude, or altitude). In Egypt, the wild *Solanum* is represented by nine species, including *S. coagulans* Forssk., *S. elaeagnifolium* Cav., *S. forskalii* Dunal, *S. incanum* L., *S. nigrum* L., *S. schimperianum* Hochst., *S. sinaicum* Boiss., *S. villosum* Mill., and *S. virginianum* L [41, 42]. Shaheen, et al. [43] added *S. diphyllum* L. as a new record to Egypt. Later, Särkinen, et al. [44] reported, in their revision of Old World black nightshades (Morelloid clade of *Solanum*), the occurrence of three more species (*S. memphiticum* J.F.Gmel., *S. scabrum* Mill., and *S. tarderemotum* Bitter), increasing the total number of wild *Solanum* to 13 species. However, a recent annotated checklist of the Flora of Egypt reported 11 species, 2 subspecies, and 3 varieties of *Solanum* [45]. Moreover, Hepper [46] has reported 24 introduced, cultivated species of *Solanum* in Egypt.

Localized studies of alien plant species outside their native distributional range are crucial for understanding their traits and habitat preferences [47]. Under changing climate scenarios, investigating their behavior across various ecological contexts and their capacity to invade distinct habitats gives insights for forecasting climatically favorable regions for future invasions [48]. Exploring the mechanisms behind biological invasions and the phenotypic plasticity of these taxa provides baseline data to implement region-specific strategies for long-term monitoring and prevention plans [49–51]. Additionally, descriptive studies help policymakers to implement conservation strategies to protect native biodiversity [52].

Thus, the current study integrated macro- and micro-morphology, scanning electron microscopy (SEM) of seeds and pollen grains, histological anatomy of vegetative organs, and multi-locus phylogenetic analysis to provide a thorough taxonomic characterization of the poorly described *S. argentinum*. This study provides the first documented record of *S. argentinum* in Egypt, which represents a significant geographical expansion for this woody species. We documented the historical presence of the species (over 60 years) by re-evaluating the CAI and Kew historical sheets (collected in December 1963). Furthermore, assessingphenotypic variation of the newly reported species will facilitate its identification, monitoring, and tracking of potential escape or naturalization.

## Materials and methods

### Plant material

*S. argentinum* specimens were collected from the Giza Zoo, Giza Governorate, Egypt (lat. 30°01’40.2"N, 31°12’54.6"E) between April and August of the years 2022–2024 by Hamdy, R.S. Digital photos of the plant were taken in the field to confirm the identification. We deposited voucher specimens at the herbaria of Cairo University (CAI), Assiut University (ASTU), and Qena University (QNA) in Egypt. A complete list of the specimens examined in this study is supplied in Supplementary Table S1. Herbarium acronyms follow Thiers [53]. To identify the plant specimens, we reviewed the digital sources [1], World Flora Online [54], Solanaceae Source (http://solanaceaesource.org) [3], Floras of Egypt and the nearby countries, and related literature [6, 36–38, 40].

### Macro- and micro-morphological examinations

Representative fresh samples of vegetative parts (stems, petioles, and leaves) and reproductive parts (buds, flowers, and fruits) were fixed in FAA solution (formalin: acetic acid: 50% alcohol; 1:1:18 v/v) for 48 h and stored in 70% ethanol for further examinations. Morphological examinations, measurements, and dissection of flowers were performed using an Olympus SZ61 stereomicroscope coupled with an Olympus SC100 digital camera. For light microscopy, we used an Olympus CX41 microscope equipped with an Olympus SC30 digital camera (Olympus, Germany). For scanning electron microscopy, samples were prepared as described by Mohamed, et al. [55] and examined with a TM3000 miniscope (Hitachi High-Tech). Four replicates from five different individuals were selected for the LM and SEM analyses (*n* = 20) as indicated in Table 1.

**Table 1:** Quantitative morphological characteristics of vegetative and floral organs of *Solanum argentinum* in Egypt. Values represent means ± SD of replicates based on 20 measurements per character (*n* = 20). Pollen polar axis and equatorial diameter measurements were based on 30 randomly selected grains (*n* = 30)

Character	Minimum	Maximum	Mean ± SD
Stem diameter	0.20	0.60	0.4 ± 0.15
Leaf length (cm)	17.50	3.00	12.0 ± 4.57
Leaf width (cm)	5.70	1.50	4.0 ± 1.37
Petiole length (cm)	0.30	2.20	1.2 ± 0.65
Stomata length (µm)	22.00	30.00	25.7 ± 2.91
Stomata width (µm)	15.00	25.00	19.6 ± 3.21
Stomatal density (per 1 mm^2^)	165.34	203.49	186.0 ± 13.14
Epidermal cell density (per 1 mm^2^)	483.30	546.89	515.0 ± 23.79
Stomatal index (%)	23.21	28.30	26.4 ± 1.86
Calyx length (mm)	1.20	1.87	1.5 ± 0.19
Calyx width (mm)	0.53	0.72	0.6 ± 0.07
Calyx teeth (mm)	0.50	1.00	0.66 ± 0.16
Calyx teeth/calyx length (%)	33.00	46.00	0.4 ± 0.06
Corolla length (mm)	3.39	4.26	3.7 ± 0.27
Corolla width (mm)	1.28	1.98	1.6 ± 0.25
Stamen length (mm)	2.00	2.50	2.2 ± 0.18
Anther length (mm)	1.50	2.00	1.65 ± 0.17
Anther width (mm)	0.38	0.63	0.5 ± 0.10
Filament tube length (mm)	0.45	0.65	0.55 ± 0.07
Pollen polar axis (P)(µm)	20.00	25.00	21.8 ± 1.25
Pollen equatorial diameter (E)(µm)	12.00	16.00	13.3 ± 0.92
P/E (%)	147.00	200.00	1.6 ± 0.14
Stigma length (mm)	0.75	1.00	0.87 ± 0.10
Style length (mm)	2.70	3.00	2.9 ± 0.11
Style width (mm)	0.18	0.30	0.23 ± 0.05
Fruit length (mm)	3.50	6.00	4.3 ± 0.86
Fruit width (mm)	3.50	6.00	4.4 ± 0.78
Seed length (mm)	2.27	2.96	2.7 ± 0.20
Seed width (mm)	2.69	3.45	3.0 ± 0.23

Trichomes of various vegetative parts (adaxial and abaxial leaf surfaces, stems, peduncles) and floral parts (calyx and corolla) were observed and documented using both light microscopy and scanning electron microscopy. The terminology used to describe the trichome types followed Roe [56], and Katinas [57]. For stomata measurements, epidermal peels of leaves were prepared and examined under 200× and 400× magnifications using light microscopy. We counted stomata, epidermal cell densities, and stomatal index per unit area, following the method described by Hassan, et al. [58]. Measurements were based on 10 fields of view from three different leaves of five *Solanum* individuals.

Pollen grains were acetolysed following Erdman [59], then stained with Safranin (1% Safranin solution in 50% ethanol) and mounted in glycerin jelly before light microscopy examination. Pollen grains were detached from fertile anthers and directly examined without acetolysis for scanning electron microscopy. Pollen measurements were based on 30 randomly selected grains (*n* = 30). We selected fully ripened seeds and examined them by light and scanning microscopy as described in Aboulela, et al. [60] and Saleh, et al. [61]. Seed measurements were based on four mature seeds from five different individuals.

### Anatomical examinations

Histological sections (stem, petiole, and lamina) prepared from three *S. argentinum* individuals were embedded in paraffin wax and cut at 10–15 μm using a Reichert sliding microtome (Austria) as described by Johansen [62]. Twelve sections per organ (6 sections × 2 slides) were examined under a light microscope, amounting to 36 sections across the three organs (stem, petiole, and leaf) per individual. We prepared permanent slides by dehydrating the cross sections in an alcohol series and double-stained them with safranin 50% and light green 96%. The slides were examined with an Olympus CX41 microscope and documented with an Olympus SC30 digital camera. We followed Koch, et al. [63] for the description of the shape and outlines of epidermal cells and the terminology of the International Association of Wood Anatomists (IAWA) [64], Metcalfe and Chalk [65], Kannan, et al. [66], and da Rocha, et al. [67] for the description of other anatomical traits.

### DNA extraction, PCR amplification, and gel electrophoresis

Fresh leaves were collected and preserved in silica gel, then ground in liquid nitrogen with a Multi-beads Shocker (Yasui Kikai, Osaka, Japan). Total genomic DNA was extracted using the DNeasy Plant Mini Kit (Qiagen) according to the manufacturer’s instructions, from a single individual deposited in the ASTU herbarium with the accession number (ASTU-MA1001). We assessed DNA quality using a NanoDrop 2000 spectrophotometer (Thermo Scientific, Wilmington, USA) and agarose gel electrophoresis. For polymerase chain reaction (PCR), we used a total volume of 20 µL of PCR reaction mixture containing the following: 10 µL of 2 × Quick Taq™ HS DyeMix (Toyobo), 0.4 µL of each primer, 2 µL of genomic DNA, and nuclease-free water (7.2 µL). A T100 thermal cycler (Bio-Rad, Hercules, CA, USA) was used to amplify the three markers (ITS2, *rbcL*, and *trnL*). The PCR reaction conditions were as follows: initial denaturation at 94 °C for 5 min, followed by 35 cycles of 94 °C for 30 s (denaturation), 56 °C for 30 s (annealing), and 72 °C for 60 s (extension), and a final extension at 72 °C for 10 min. The primers, ITS2-F (5`- ATG CGA TAC TTG GTG TGA AT-3`) and ITS2-R (5`- GAC GCT TCT CCA GAC TAC AAT-3`); *rbcL*-F (5`-ATG TCA CCA CAA ACA GAG ACT AAA GC -3`) and *rbcL*-R (5`- GAA ACG GTC TCT CCA ACG CAT-3`); and *trnL*-F (5`- CGA AAT CGG TAG ACG CTA CG-3`) and *trnL-*R (5`- GGG GAT AGA GGG ACT TGA AC-3`) were used to amplify the molecular markers ITS2, *rbcL*, and *trnL*, respectively. The PCR products were verified on 1.5% (w/v) TBE agarose gel electrophoresis and visualized by pre-added HydraGreen™ Safe DNA Dye under UV light.

### DNA sequencing

PCR products were purified using a FavorPrep™ Gel/PCR Purification Mini Kit (Favorgen Biotech Corp, Taiwan) and sequenced by an ABI 3500 Genetic Analyzer (Applied Biosystems, HITACHI, Japan) using a BigDye Terminator v3.1 Cycle Sequencing Kit. The DNA sequences were tentatively identified using the Basic Local Alignment Search Tool (BLASTN; https://blast.ncbi.nlm.nih.gov/Blast.cgi) algorithm available on the National Center for Biotechnology Information (NCBI) website (Zhang et al. 2000). The novel sequences of *S. argentinum* were deposited in GenBank (http://www.ncbi.nlm.nih.gov/) under the accession numbers: PZ273763, PZ302158, and PZ302159.

### Sequence alignments

We generated three new sequences of the studied species from three DNA molecular markers (ITS2, *rbcL*, and *trnL*) and edited them using Geneious^®^ 9.1.2 [68]. To place this taxon within the phylogenetic context of *Solanum*, we compared the sequences with those previously deposited in the GenBank database (http://www.ncbi.nlm.nih.gov/) using BLASTN. Wherever identical sequences of multiple accessions of the same taxon were identified (using the Finding Duplicates Plugin in Geneious 9.1.2) in any of the three data sets, only one individual sequence was used to represent the species in the final analyses. We included all sequences of *S. argentinum* and its putatively closest relatives available in GenBank for the three markers at the time of analysis in Supplementary Table S2. We aligned all sequences using the Muscle Alignment algorithm, followed by minimal manual corrections in Geneious 9.1.2 [68].

Because the single markers lacked phylogenetically informative characters and interspecific relationships within the monophyletic groups in these datasets were unclear or weakly supported, we combined the three markers to produce well-resolved, well-supported phylogenetic relationships.

The final alignment of the combined ITS2 + *rbcL* + *trnL* datasets included 59 sequences, resulting in a 1,290 bp alignment, comprising 1169 bp (90.62%) conserved sites, 59 bp (4.57%) variable sites, and 62 bp (4.81%) parsimony-informative sites (Supplementary Table S3).

### Evaluation of incongruity between the data sets

To determine if the molecular data partitions were combinable and to consider the possibility of discordant results due to laboratory errors, Incongruence Length Difference Tests (ILD: [69]) were implemented as a partition homogeneity test in PAUP* 4.0 b10 [70], excluding constant characters. Three partitions were defined and tested. We conducted parsimony heuristic searches with tree-bisection-reconnection (TBR) branch swapping and stepwise simple taxon addition, with 1000 random-addition replicates, holding five trees at each step of score ≥ 1, setting the maxtrees to 1000, and coding gaps as missing. The ILD test yielded a *p*-value of 1.000 (0 out of 1,000 replicates showing greater conflict than observed), indicating no statistically significant incongruence among the ITS2, *rbcL*, and *trnL* partitions. Inspection of individual-marker trees (Supplementary Figs. S1–S3) revealed no well-supported conflicts (i.e., no nodes with bootstrap support ≥ 70% supporting alternative relationships). Based on the combination of the non-significant ILD test and the absence of strongly supported topological conflict, the three markers were concatenated for the primary phylogenetic analysis.

### Phylogenetic analyses

Phylogenetic reconstruction was performed using Maximum Likelihood (ML) and Bayesian Inference (BI) on the concatenated data (Supplementary material S1). RAxML GUI 2.0 was used to perform ML analysis [71]. The analysis was run with the alignment partitioned by locus, using the parameters of ModelTest-NG, an implemented algorithm in RAxML GUI v.2.0.10, to select the best-fit partition model using the AICc criterion. Best-fit models, based on AICc, were HKY + I + G4 (ITS2), TPM1uf (*trnL*), and TIM1 + I (*rbcL*) for ML. Nodal support for the ML analysis was estimated using a nonparametric bootstrap validation with 1,000 pseudo-replicates.

Bayesian Inference (BI) was performed on the concatenated data using MrBayes v3.2.5 [72], with the alignment partitioned by locus. Prior to the Bayesian phylogeny analysis, we used MrModeltest v.2.3 [73] to select the best nucleotide substitution model using the Akaike information criterion (AIC) [74]. HKY + I + G4 (ITS2), GTR (*trnL*), and GTR + I (*rbcL*) models were selected. Two Markov Chain Monte Carlo (MCMC) runs were implemented in MrBayes, using four linked chains (three heated and one cold), with 2 × 10^7^ generations and trees sampled every 1000 generations. The first 25% of trees were discarded as burn-in, and consensus trees with posterior probabilities (PP) of clades were calculated from the post-burn-in trees using a 50% majority rule [75]. Convergence diagnostics, including ESS and PSRF values, are available in the MCMC log file (Supplementary material S2). The tree file was visualized and edited using FigTree v1.4.4, with *Alkekengi officinarum* Moench used as a more distant outgroup to root the phylogeny [29].

## Results

The morphological comparison of *S. argentinum* specimens with the full species description available on Solanaceae Source (http://solanaceaesource.org) and with those described by Bitter [36] and Knapp [6] has revealed the main diagnostic morphological characteristics and confirmed the identification of this species. The taxonomic treatment below summarizes the morphological and anatomical features and helps distinguish this introduced species in Egypt.

### Taxonomy

*Solanum argentinum* Bitter & Lillo, Repert. Spec. Nov. Regni Veg. 12: 547. 1913 [36]. TYPE: Argentina. Córdoba: prope Córdoba, T. Stuckert 15,164 (LECTOTYPE: designated by Knapp, 2008, CORD!; isotypes, G!, LIL).

### Heterotypic Synonyms

*Solanum argentinum* Bitter & Lillo var. *chroniotrichum* Bitter, in Repert. Spec. Nov. Regni Veg. 12: 549 (1913) [36].

*Solanum chroniotrichum* (Bitter) C. V. Morton, in Revis. Argentine Sp. *Solanum* 183 (1976) [10].

*Solanum microcarpum* Cerv. ex Lag. in Gen. Sp. Pl.: 10 (1816) [76].

### Distribution

**Fig. 1:**
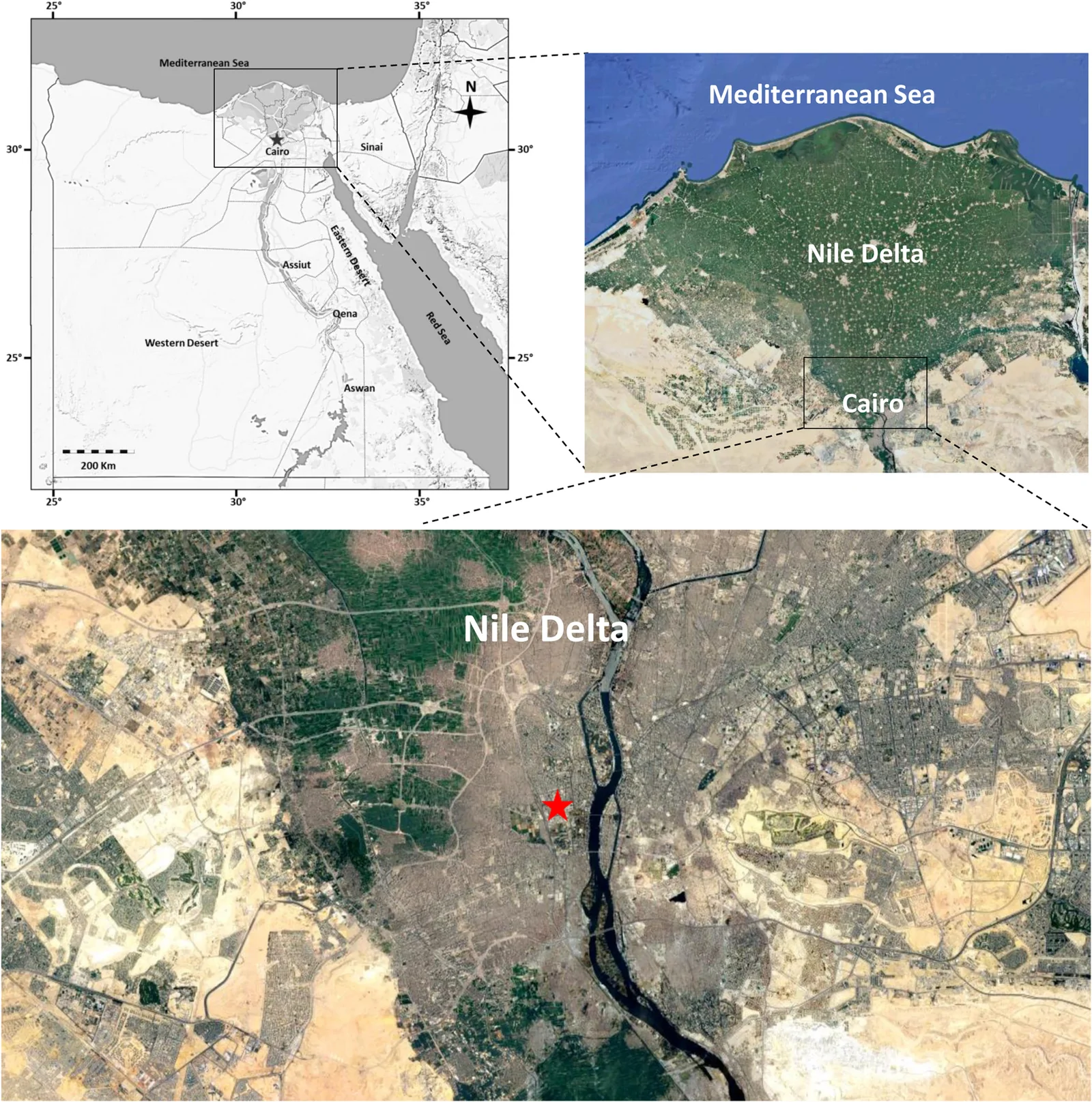
Distribution map of *Solanum argentinum* in Egypt. The star indicates the exact location of Giza Zoo, where the plant material was collected

It is a widespread shrub species native to Argentina, Bolivia, and Paraguay. The present study revealed the occurrence of *S. argentinum* in Egypt. Plant materials were collected from a single site inside the Giza Zoo, as shown in the distribution map (Fig. 1).

### Phenology and conservation status

Based on our field observations and the records of the Egyptian herbaria, flowering of *S. argentinum* occurs between July and October, with peak flowering in August, while the plant bears fruit over a broader period, between September and April.

*S. argentinum* was introduced to Egypt approximately 60 years ago and has been recorded mainly as an ornamental plant within the zoological garden under irrigated conditions. Field observations by Hamdy RS from 2022 to 2024 confirmed its continued presence and successful growth. The studied population was estimated at approximately 150 individuals, although no national-scale population census has been conducted. Its occurrence outside the zoo remains insufficiently documented to determine its wider distribution or future range expansion. Currently, the plant status in Egypt is best regarded as an introduced species with a localized occurrence. According to regional applications of the IUCN Red List criteria and institutional biodiversity databases, the preliminary conservation status of *S. argentinum* is evaluated as Least Concern (LC) because it is a widespread, highly adaptable plant species [54]. However, a formal IUCN conservation status assessment for Egypt is currently premature, and we recommend the necessity of continuous monitoring to track its future survival and potential ecological risks.

### Macro- and micro-morphological features

**Fig. 2:**
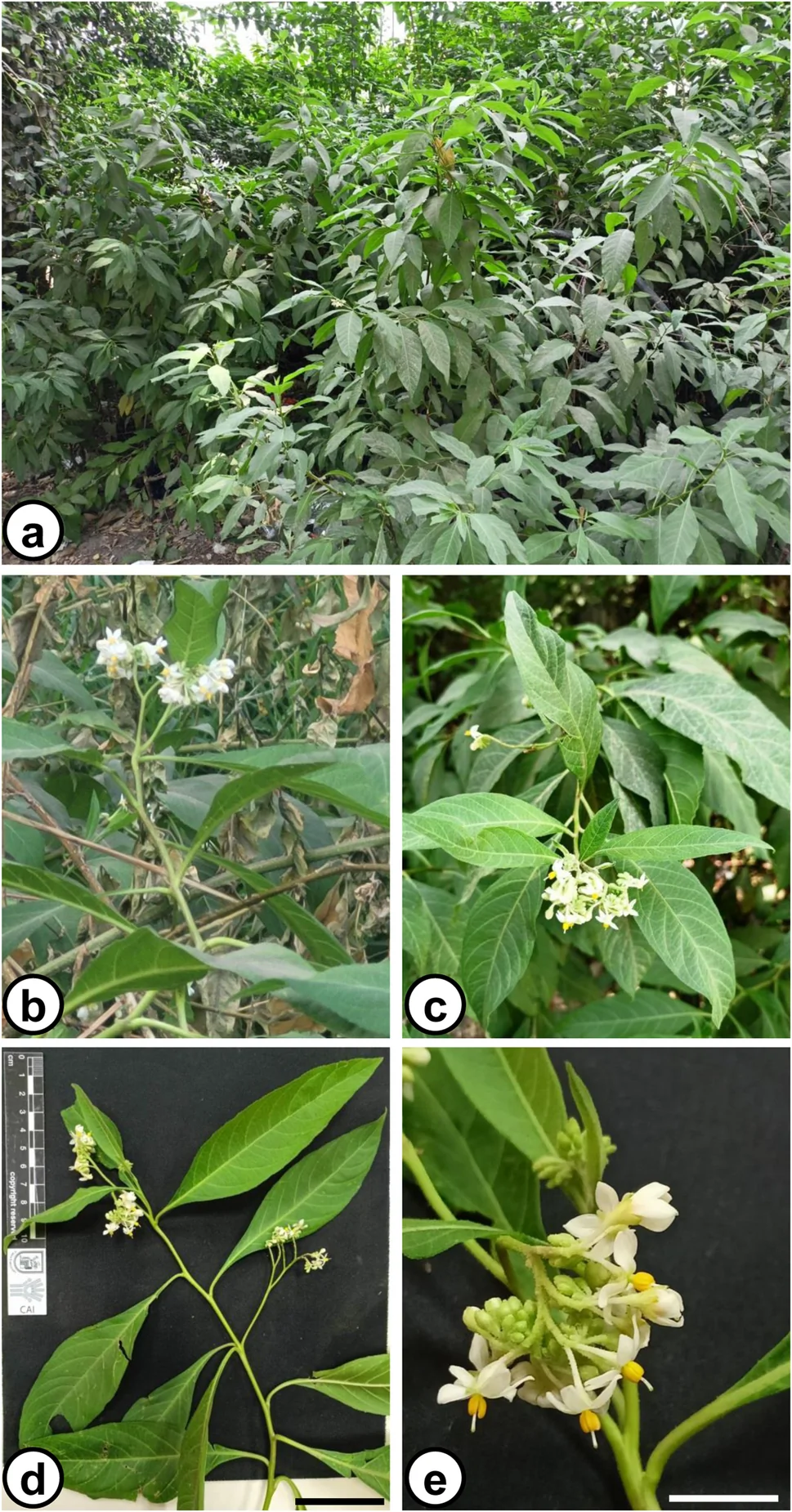
Habit of *Solanum argentinum. ***a**–**c** photos in the field; (**d**, **e**) photos in the lab. **a** showing the habit of the plant. **b**–**d** showing a vegetative part of the plant with a flowering shoot. **e** showing a many-times-branched inflorescence with multiple flowers. Scale bars = 5 cm (**d**), 2 cm (**e**)

*S. argentinum* is a shrub up to 2 m high. Stems terete, erect, ca. 0.2–0.7 cm in diameter, sparsely to densely pubescent, woody, with dark brown bark near the base; young branches were prominently green. Leaves simple, alternate, exstipulate; petiole (0.4–2.2) cm long, glabrous to densely pubescent, prominently green; lamina 3–17.5 cm × 1.5–5.7 cm, elliptic to narrowly elliptic, symmetric, sparsely to densely pubescent, base attenuate, margins entire, apex acute or acuminate (Fig. 2; Table 1).

**Fig. 3:**
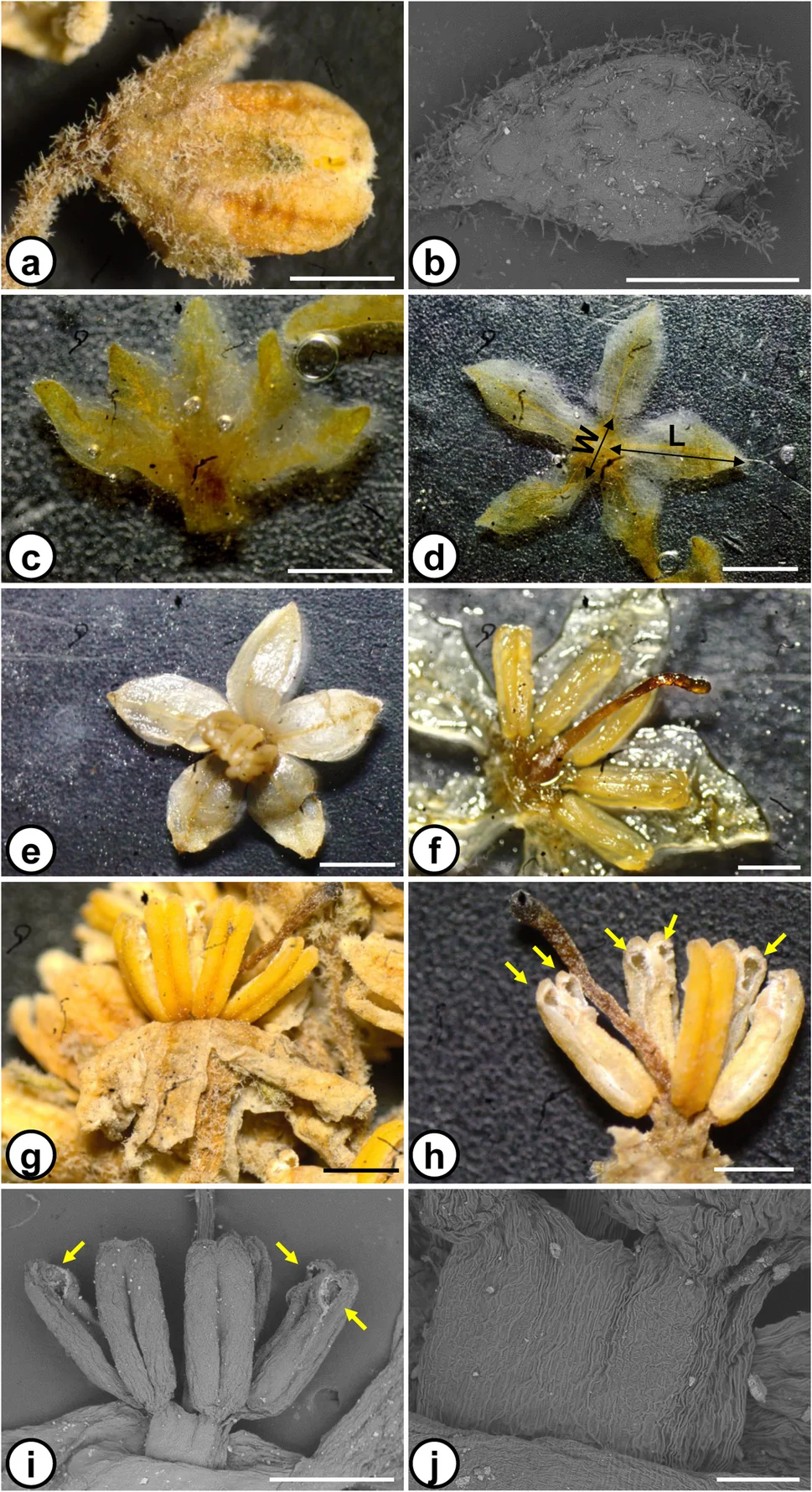
Morphological characteristics of *Solanum argentinum* flowers. **a**, **b** floral bud, the sepals are densely covered by bicellular furcate and dendritic non-glandular trichomes and sparsely covered with unicellular non-glandular and uniseriate glandular trichomes with unicellular head; (**c**) sepals of an open flower; (**d**, **e**) petals of an open flower; (**f**) stamens, style, and stigma of an open flower; (**g**, **h**, **I**, **j**) stamens. The arrows in (**d**) indicate how we measured the length (L) and width (W) of the corolla. Note that the five stamens are connate at the filaments, forming a short tube (**h**, **I**, **j**). Also note the poricidal pores at each anther locule to discharge the pollen grains (arrows in **h**, **i**). (**a**, **c**, **d**, **e**, **f**, **g**, **h**) Light microscopy micrographs; (**b**, **i**, **j**) SEM micrographs. Scale bars = 2 mm (**d**, **e**), 1 mm (**a**, **b**, **c**, **f**, **g**, **h**, **i**), 200 μm (**j**)

Inflorescences are internodal or terminal, multi-branched, with ca. 50–200 flowers, sparsely to densely pubescent with dendritic trichomes, aggregated on a common peduncle, 5.3–7.2 cm; flowers are ebracteate, pedicels 0.6–0.9 cm. Calyx densely pubescent, 1.2–1.9 cm long, 0.5–0.7 cm wide (Fig. 3; Table 1), teeth (lobes) 0.5–0.9 mm, narrowly triangular, divided to one-third to less than halfway (33–46%), sparsely dendritic pubescent abaxially. Corolla white, stellate in shape, radially symmetric (actinomorphic), glabrous to densely papillate toward the apex, 3.4–4.2 cm in length, and 1.3–2 cm in width, lobed ca. 3/4 of the way to the base, the lobes 0.4–0.75 cm, deltate, flat to slightly reflexed at anthesis. Stamens 2.0–2.5 cm in length, glabrous, forming a staminal column (Fig. 3; Table 1); filaments connate, forming a short tube, 0.45–0.65 mm in length, and glabrous (Fig. 3h, i, j; Table 1); anthers 2–2.5 × 0.4–0.65 mm, yellow, elliptic-oblong in shape, poricidal at the tips, the pores slightly elongating to short tear-drop-shaped slits at anthesis (arrows in Fig. 3h, i, j). The gynoecium consisted of two fused carpels with oblique axile placentation; the ovary conical, glabrous; style 2.7–3 mm long, cylindrical, straight, glabrous; stigma clavate, 0.75–1.0 mm long.

**Fig. 4:**
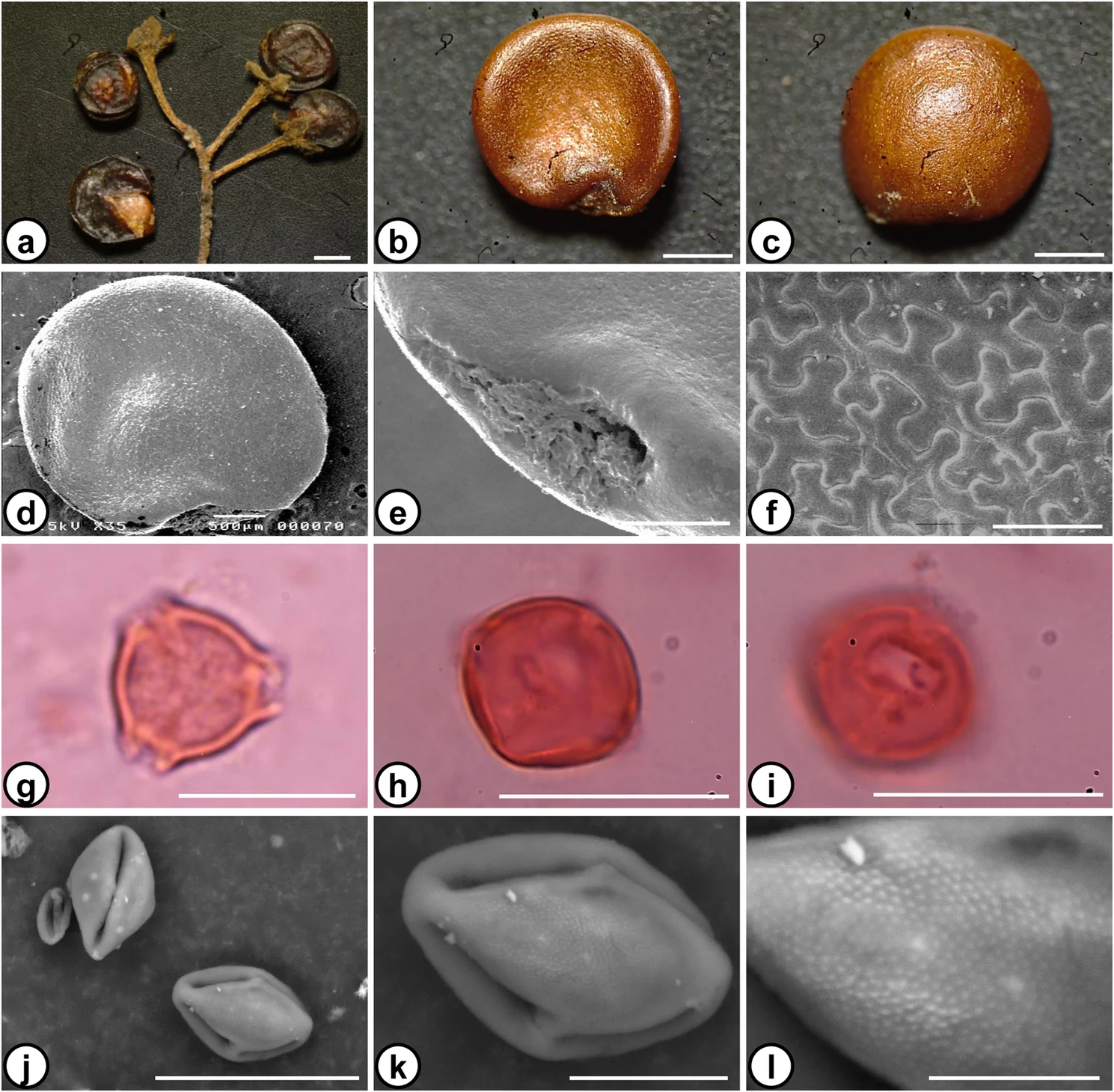
Fruits, seeds, and pollen grains of *Solanum argentinum. ***a** node of a fruiting branch bearing fruits; (**b**, **c**) stereo-micrographs of seed in lateral views; (**d**-**f**) SEM micrographs of seed showing outline (**d**), hilum (**e**), and surface ornamentation (**f**); (**g**-**i**) LM micrographs of pollen grains in polar view (**g**) and equatorial view (**h**, **i**). Note the compound aperture (lalongate pore) below the colpus (**i**); (**j**-**l**) SEM micrographs of pollen grains in equatorial view. Scale bars = 2 mm (**a**), 500 μm (**d**, **e**), 100 μm (**b**, **c**), 50 μm (**f**), 30 μm (**j**), 20 μm (**g**, **h**, **i**), 10 μm (**k**), 5 μm (**l**)

Fruit is a globose berry, 3.5–6 mm in height × 3.5–6 mm in diameter, yellowish-orange or bright orange in color at maturity, turning reddish-brown after drying, glabrous, shiny; fruiting pedicels are 7–9 mm long, 1.8–2.2 mm in diameter at the base, swollen just below the berry (Fig. 4; Table 1). Seeds 4–8 per berry, yellowish-brown in color, shiny, ca. 2.7–3.5 × 2.2–3 mm, flattened to slightly convex reniform, hilum terminal, ovate in shape (Fig. 4b–e). The seed surface was minutely pitted, with irregularly reticulate ornamentation (Fig. 4f). The anticlinal walls were thin, grooved, and strongly undulated, and the periclinal walls were smooth, slightly concave (Fig. 4f).

### Palynology

Pollen grains were tricolporate with a compound aperture consisting of a lalongate pore below the colpus (Fig. 4g–l). They were prolate in shape (P/E = 1.47–2) with an obtuse triangular outline in polar view and an obtuse rhombic outline in equatorial view. The pollen size was small (20–25 × 12–16 μm) (Fig. 4; Table 1). The surface ornamentation was scabrate (Fig. 4k, l).

### Indumentum and stomatal complexes

**Fig. 5:**
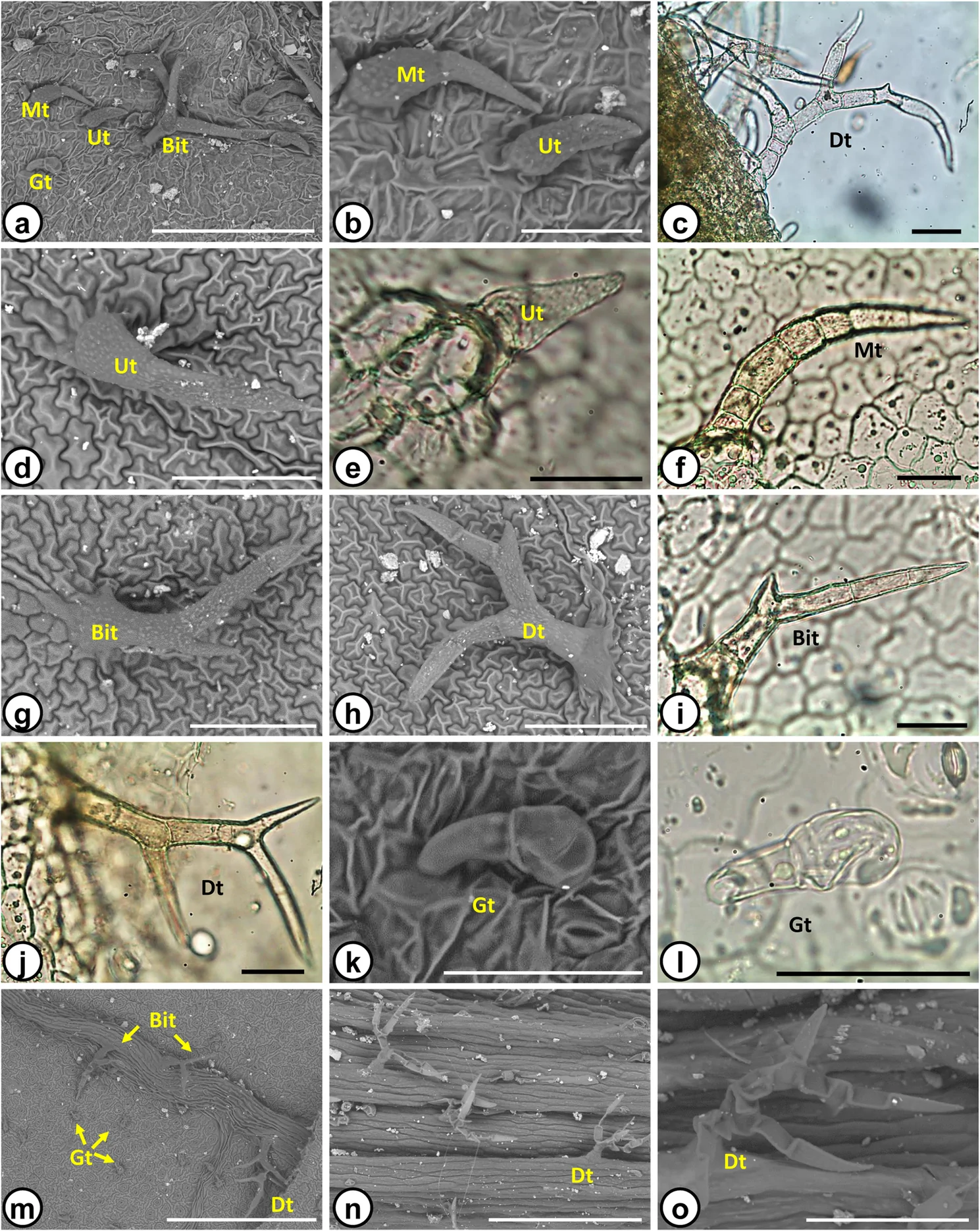
Indumentum of *Solanum argentinum. ***a**-**c** calyx; (**d**-**j**) adaxial leaf surface; (**k**-**m**) abaxial leaf surface; (**n**, **o**) stem. (**a**, **b**, **d**, **g**, **h**, **k**, **m**, **n**, **o**) SEM micrographs; (**c**, **e**, **f**, **I**, **j**, **l**) light microscopy micrographs. Note that the calyx is covered with unicellular, multicellular uniseriate, bicellular furcate, and dendritic non-glandular trichomes as well as glandular trichomes. The adaxial leaf surface contains unicellular, multicellular uniseriate, bicellular furcate, and dendritic non-glandular trichomes over the entire surface. In contrast, these types of trichomes are confined to the veins on the abaxial leaf surface. The abaxial leaf surface is also covered with glandular trichomes. The trichomes on the stem are mainly dendritic. Cuticular warts are present on the surface of the non-glandular trichomes and absent on the surface of the glandular ones. Bit, bicellular furcate trichome;; Dt, dendritic trichome; Gt, glandular trichome; Mt, multicellular uniseriate trichome; Ut, Unicellular trichome. Scale bars = 500 μm (m), 300 μm (**n**), 200 μm (**a**), 100 μm (**d**, **g**, **h**, **o**), 50 μm (**b**, **c**, **e**, **f**, **i**, **j**, **k**, **l**)

Light and scanning electron microscopy observations revealed that *S. argentinum* had an interesting indumentum. The trichome distribution varied across vegetative and floral organs, including the calyx, pedicel, stem, and adaxial and abaxial leaf surfaces. We recorded the occurrence of both glandular and non-glandular trichomes on these plant parts. Non-glandular trichomes were more diverse than the glandular types. The non-glandular trichomes included unicellular, multicellular uniseriate, bicellular furcate, and dendritic trichomes (Fig. 5), whereas, the glandular trichomes were all of one type (uniseriate with a unicellular head). Cuticular warts were observed on the surface of the non-glandular trichomes (Fig. 5).

In contrast, such warts were absent from the surface of the glandular ones (Fig. 5a–j). The pedicel and calyx contained a mixture of all recorded glandular and non-glandular trichome types (Fig. 5a–c). Interestingly, the adaxial and the abaxial leaf surfaces contained different distributions of the trichomes. Unicellular, multicellular uniseriate, bicellular furcate, and dendritic non-glandular trichomes were observed over the entire adaxial leaf surface (Fig. 5d–j), whereas these types of trichomes are confined to the veins of the abaxial surface (Fig. 5k–m). Moreover, the abaxial leaf surface was entirely covered with uniseriate glandular trichomes with unicellular heads, whereas the adaxial leaf surface was devoid of this trichome type (Fig. 5k–m). The stem was covered with both glandular (uniseriate with unicellular heads) and non-glandular trichomes (mainly dendritic) (Fig. 5n, o).

**Fig. 6:**
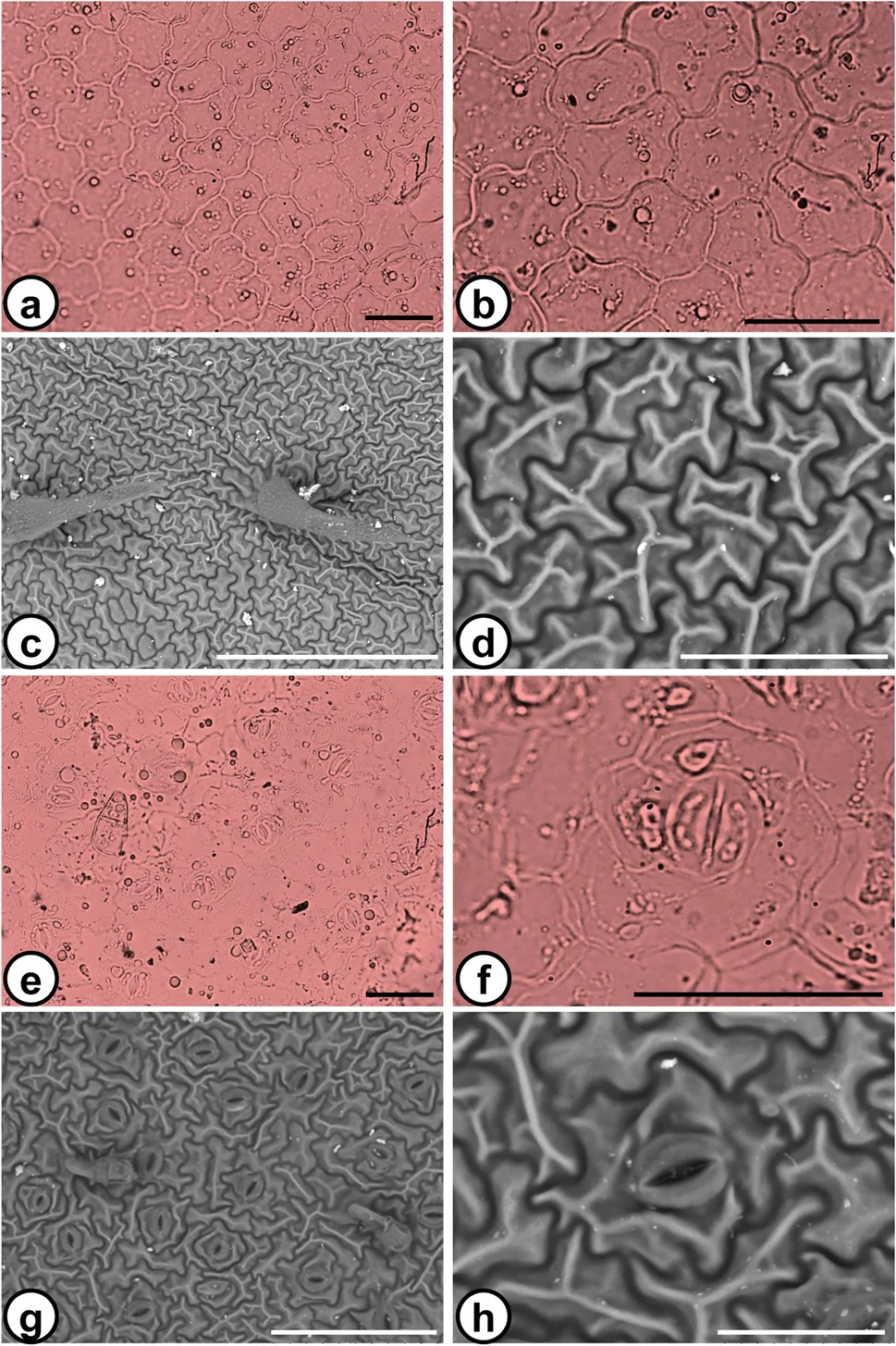
Leaf micro-morphology of *Solanum argentinum. ***a**-**d** adaxial surface; (**e**-**h**) abaxial surface. **a**, **b**, **e**, **f** LM micrographs; (**c**, **d**, **g**, **h**) SEM micrographs. Note that the adaxial leaf surface contains no stomata. Glandular trichomes are found only on the abaxial leaf surface. Scale bars = 50 μm (**a**, **b**, **d**, **e**,** f**), 200 μm (**c**), 100 μm (**g**), 30 μm (**h**)

We described the characteristics of stomata and epidermal cells on both the adaxial and abaxial leaf surfaces (including their types, distribution, size, and density). Stomata of *S. argentinum* were hypostomatic (present solely on one side of the leaf; the abaxial). The adaxial leaf surface was devoid of stomata and contained only polygonal epidermal cells with U-undulated cell boundaries and sunken anticlines (Fig. 6a–d). The stomata on the abaxial leaf surface were mainly of the anisocytic type, while a few were of the anomocytic type (Fig. 6e–h). The stomata ranged in length from 22 to 30 μm and in width from 15 to 25 μm. The average stomatal density on the abaxial leaf surface was 186 per mm^2^, and the average epidermal cell density was 515 per mm^2^, whereas the stomatal index was 26.4%.

### Anatomical features

#### Stem

**Fig. 7:**
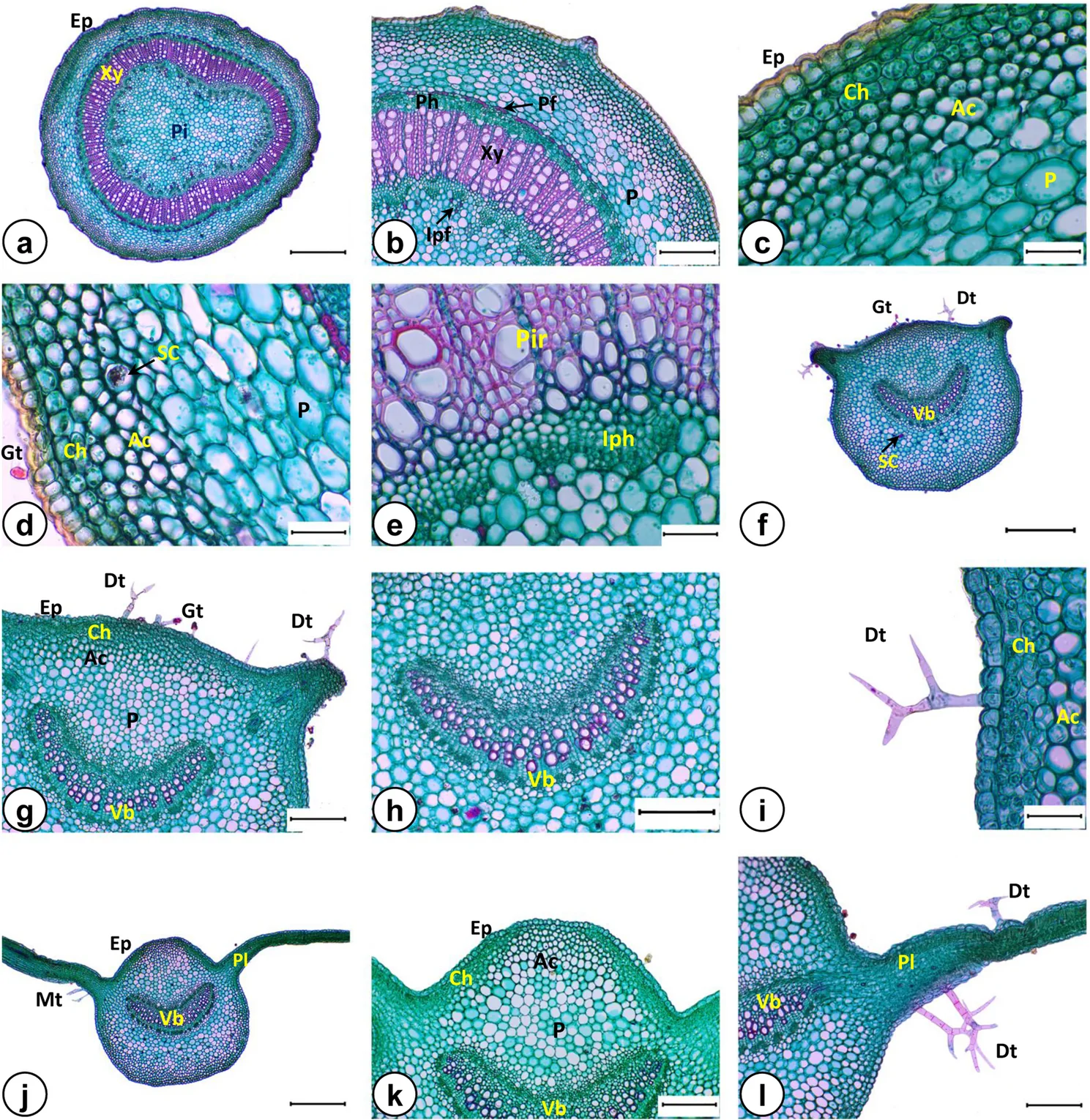
Cross sections of *Solanum argentinum*. **a–e** stem (**a.** general outline, **b.**,** c.**,** d.**,** e.** enlarged views); **f–i** petiole (**f.** general outline, **g.**,** h.**,** i.** enlarged views); **j–l** leaf (**j.** general outline, **k**., **l.** enlarged views). [Ac. angular collenchyma; Ch. chlorenchyma; Dt. dendritic trichome; Ep. epidermis; Gt. glandular trichome; Iph. intraxylary phloem; Ipf. inner pericyclic fibers; Mt. multicellular uniseriate trichome; P. parenchyma; Pf. pericyclic fibers; Ph. phloem; Pi. pith; Pir. pith ray; Pl. palisade tissue; Sc. sand crystal; Vb. vascular bundle; Xy. xylem]. Scale bars = 500 μm (**a**, **f**, **j**), 200 μm (**b**, **g**, **h**, **k**, **l**), 50 μm (**c**, **d**, **e**, **i**)

The stem outline was circular in cross-section. The stem was surrounded by a single compactly arranged layer of epidermal cells with convex outlines. The epidermal cells were covered with a thin layer of cuticle and contained stomata, glandular trichomes, and dendritic non-glandular trichomes (Fig. 7a–d). The cortex consisted of 2–3 layers of chlorenchyma cells, followed by 2–4 layers of angular collenchyma cells, and then 6–9 layers of parenchyma cells (Fig. 7b–d). Most parenchymatous cells were spherical, while a few were polyhedral. The vascular system of the stem consisted of bicollateral vascular bundles (Fig. 7a, b, e). The secondary phloem fibers were composed of patches of 1–2 layers of sclerenchyma cells. The cambium cells were distinguishable by 4–6 compacted layers. Xylem was made of a nearly continuous ring intersected with primary uni- to bi-seriate pith rays (Fig. 7e). Xylem vessels were arranged in radial multiples with diffuse-porous patterns. The xylem vessels were partly solitary and partly in radial multiples of 2–4 s. The solitary vessel outline was angular (Fig. 7b, e). Intraxylary phloem develops via internal cambium from pith cells. Pith cells were thin-walled polyangular parenchyma cells (Fig. 7a, b). Sand crystals were seen through the parenchyma of the cortex and the pith (Fig. 7a, b, d).

#### Petiole

The petiole outline was circular in cross-section. It was surrounded by a single layer of globose to elongated epidermal cells covered with a thin cuticle and provided with uniseriate with unicellular head glandular trichomes and dendritic non-glandular trichomes (Fig. 7f-i). The cortex was composed of 2–4 layers of chlorenchyma, 3–5 layers of angular collenchyma, and 10–20 layers of parenchyma (Fig. 7g, i). The vascular system was composed of only one central, arc-shaped vascular bundle. Phloem was found towards both the abaxial and adaxial surfaces, whereas xylem occurred in between. Pericyclic fibers were present above the phloem of the vascular bundle (Fig. 7g, h). Sand crystals were seen within the parenchyma cells (Fig. 7f, h).

#### Lamina

The epidermis of the adaxial and abaxial surfaces was composed of one layer of tangentially elongated cells covered with a thin cuticle layer. The types and distribution of trichomes and stomata varied on both surfaces (as described above). The leaf lamina contained mesophyll of one layer of palisade cells followed by spongy parenchyma (Fig. 7j–l). The midrib region of the leaf had a single epidermis layer followed by 4–6 angular collenchyma layers at both the adaxial and abaxial surfaces. Below the angular collenchyma zone, 7–10 spongy parenchyma layers were observed. At the junctions between the lamina and mid-rib, the angular collenchyma was replaced by 4–6 layers of palisade chlorenchyma. The vascular system was very similar to that of the petiole. It contained a single, arc-shaped, bicollateral vascular bundle. Pericyclic fibers were present as separated patches of sclerenchyma above the vascular bundle (Fig. 7j, k).

### Phylogenetic analyses

BLASTN searches confirmed that all sequences belong to *Solanum* section *Geminata*, with top hits showing ≥ 98% identity for *trnL* and *rbcL*, and 100% identity for ITS2 to *S. argentinum* (Supplementary Table S2). Phylogenetic analyses used 59 combined sequences of three markers (ITS2 + *rbcL* + *trnL*) from 24 *Solanum* species and an outgroup, including three newly generated *Solanum* sequences and 56 from GenBank (Table 2), with *Alkekengi officinarum* as the outgroup [29].

**Table 2:** GenBank accession numbers for taxa included in the phylogenetic analyses of *Solanum*

Taxon	GenBank Accession Number			
	rITS2	trnL	*rbcL*	Reference
*Solanum abutiloides* Bitter & Lillo	AF244716	AY266236	—	(Bohs, 2004; Bohs and Olmstead, 2001) [77, 78]
*Solanum agrarium* Sendtn.	GU591055	GU590996	—	(Stern et al., 2011) [33]
*Solanum alternatopinnatum* Steud.	HQ457395	HQ457404	—	(Stern et al., 2011) [33]
*Solanum aphyodendron* S.Knapp	MW444627	MW447239	OP710555^^^	(Gagnon et al., 2022) [23]
*Solanum arboreum* Humb. & Bonpl. ex Dunal	AF244719	DQ180424	—	(Bohs and Olmstead, 2001; Weese and Bohs, 2007) [29, 77]
*Solanum argentinum* Bitter & Lillo	AF244718	DQ180425	—	(Bohs and Olmstead, 2001; Weese and Bohs, 2007) [29, 77]
*Solanum argentinum* Bitter & Lillo	**PZ273763**	**PZ302159**	**PZ302158**	**This study**
*Solanum cordovense* Sessé & Moc.	AF244717	DQ180480	JQ594150^^^	(Bohs and Olmstead, 2001; Weese and Bohs, 2007) [29, 77]
*Solanum gardneri* Sendtn.	GU591070	GU591015	—	(Stern et al., 2011) [33]
*Solanum humboldtianum* Granados-Tochoy & S.Knapp	MW444605	MW447213	—	(Gagnon et al., 2022) [23]
*Solanum microphyllum* Dunal	AY996529	GU591026	—	(Levin et al., 2006; Stern et al., 2011) [27, 33]
*Solanum nutans* Ruiz & Pav.	MW444628	OK087463	—	(Gagnon et al., 2022) [23]
*Solanum pseudocapsicum* L.	AF244720	AY266241	HM850368	(Bohs, 2004; Bohs and Olmstead, 2001; Schaefer et al., 2011) [77–79]
*Solanum ramonense* C.V.Morton & Standl.	MW444638	MW447252	JQ594556^^^	(Gagnon et al., 2022) [23]
*Solanum refractum* Hook. & Arn.	AY996547	HQ457408	—	(Levin et al., 2006; Stern et al., 2011) [27]
*Solanum sagittantherum* Granados-Tochoy & C.I.Orozco	MW444644	MW447262	—	(Gagnon et al., 2022) [23]
*Solanum schomburgkii* Sendtn.	GU591092	GU591042	—	(Stern et al., 2011) [33]
*Solanum sessile* Ruiz & Pav.	MW444646	MW447269	—	(Gagnon et al., 2022) [23]
*Solanum spirale* Roxb.	MH718339	MW447278	JF944462	(Gagnon et al., 2022; Li et al., 2011; Prommanee and Pamonsinlapatham, 2018) [23, 80, 81]
*Solanum talarense* Svenson	GU591096	GU591046	MZ221863	(Gagnon et al., 2022; Stern et al., 2011) [23, 33]
*Solanum tetramerum* Dunal	GU591098	GU591048	—	(Stern et al., 2011) [33]
*Solanum urticans* Dunal	GU591103	GU591051	—	(Stern et al., 2011) [33]
*Solanum wendlandii* Hook.f.	AF244731	DQ180440	—	(Bohs and Olmstead, 2001; Weese and Bohs, 2007) [29, 77]
*Solanum wrightii* Benth.	GQ480731	GQ480732	MH722386^^^	(Stern et al., 2011) [33]
Outgroup				
* Alkekengi officinarum* Moench^*^	AY665850	DQ180420	U08617	(Olmstead and Sweere 1994; Whitson and Manos 2005; Weese and Bohs 2007) (Olmstead and Sweere, 1994; Weese and Bohs, 2007; Whitson and Manos, 2005) [31, 77, 82]

^^^ The reference is an unpublished sequence from GenBank

^*^ Registered in GenBank as *Physalis alkekengi* L

The bold text indicates the newly submitted accession numbers of *S. argentinum* in this study

**Fig. 8:**
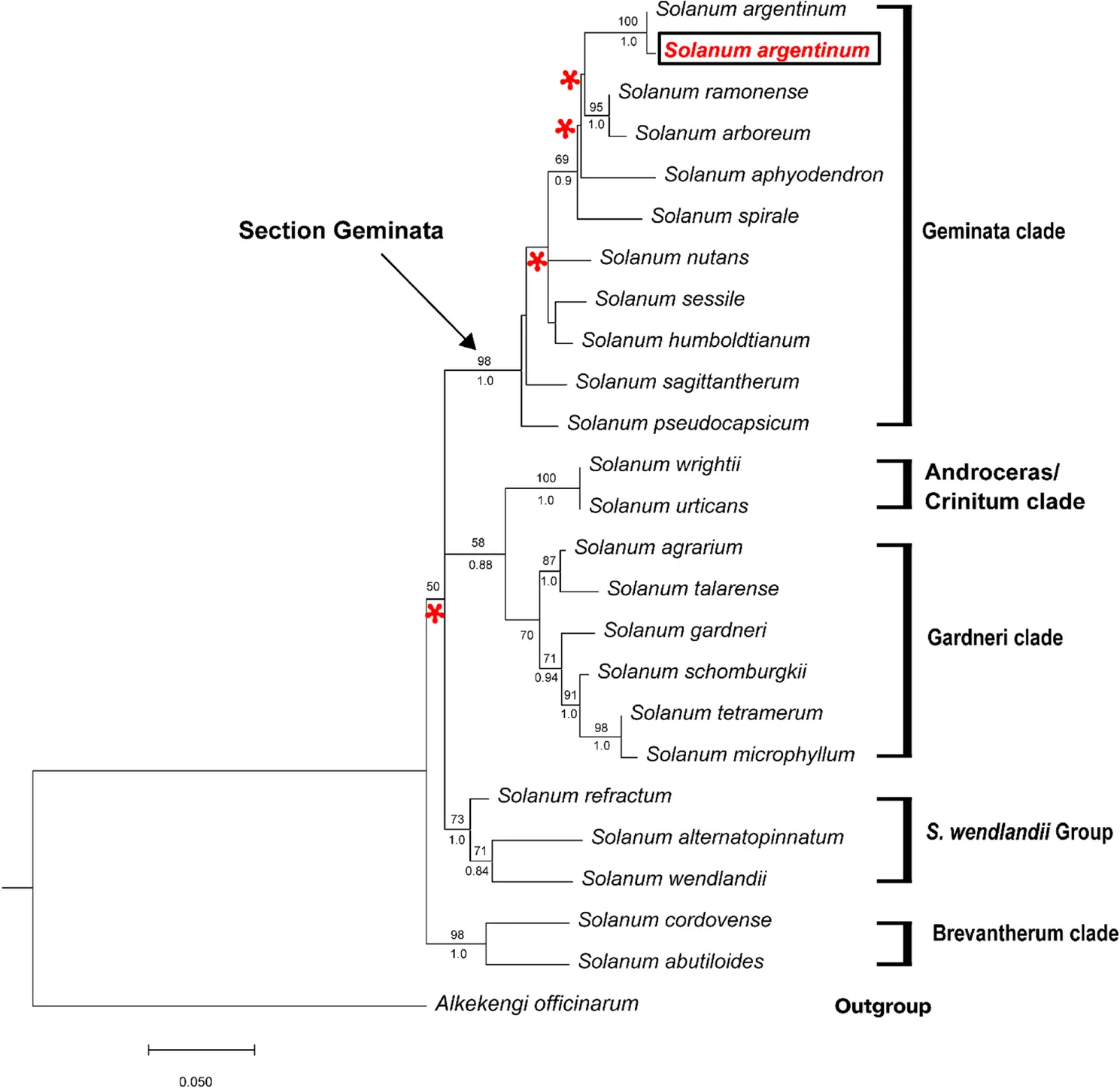
Maximum likelihood phylogenetic tree of *Solanum* section *Geminata* and outgroups based on a partitioned alignment of ITS2 and plastid *rbcL* and *trnL*. Numbers above branches are bootstrap values > 50%, followed by Bayesian posterior probabilities (only when ≥ 0.90) given below the branches. Branches marked by an asterisk collapse in the Bayesian 50% majority rule tree. The bold and boxed *Solanum argentinum* indicates the newly sequenced Egyptian material. GenBank sequences are shown in black italics. Clade affiliations are indicated. The accession numbers of the taxa included in the phylogenetic tree are listed in Table 2

Molecular identification was based on a single Egyptian specimen, which is sufficient for species-level confirmation of a first country record. The species under study forms a strongly supported clade (100% ML/1.0 BI) with *S. argentinum* and groups within the Geminata clade alongside other species (98% ML/1.0 BI; Fig. 8).

## Discussion

The documentation of *S. argentinum* in Egypt represents a significant geographical expansion for a species previously considered strictly native to Argentina, Bolivia, and Paraguay. To support the identification of our taxon as *S. argentinum*, we performed a rigorous comparison of the collected taxon from Egypt with the digital herbarium records (Kew herbarium, https://www.kew.org; POWO, 2026; WFO, 2026; Solanaceae Source website, http://solanaceaesource.org) and related literature [6, 36]. Identifying the species on a new continent is intriguing and suggests that its occurrence is likely tied to human-mediated movement.

**Fig. 9:**
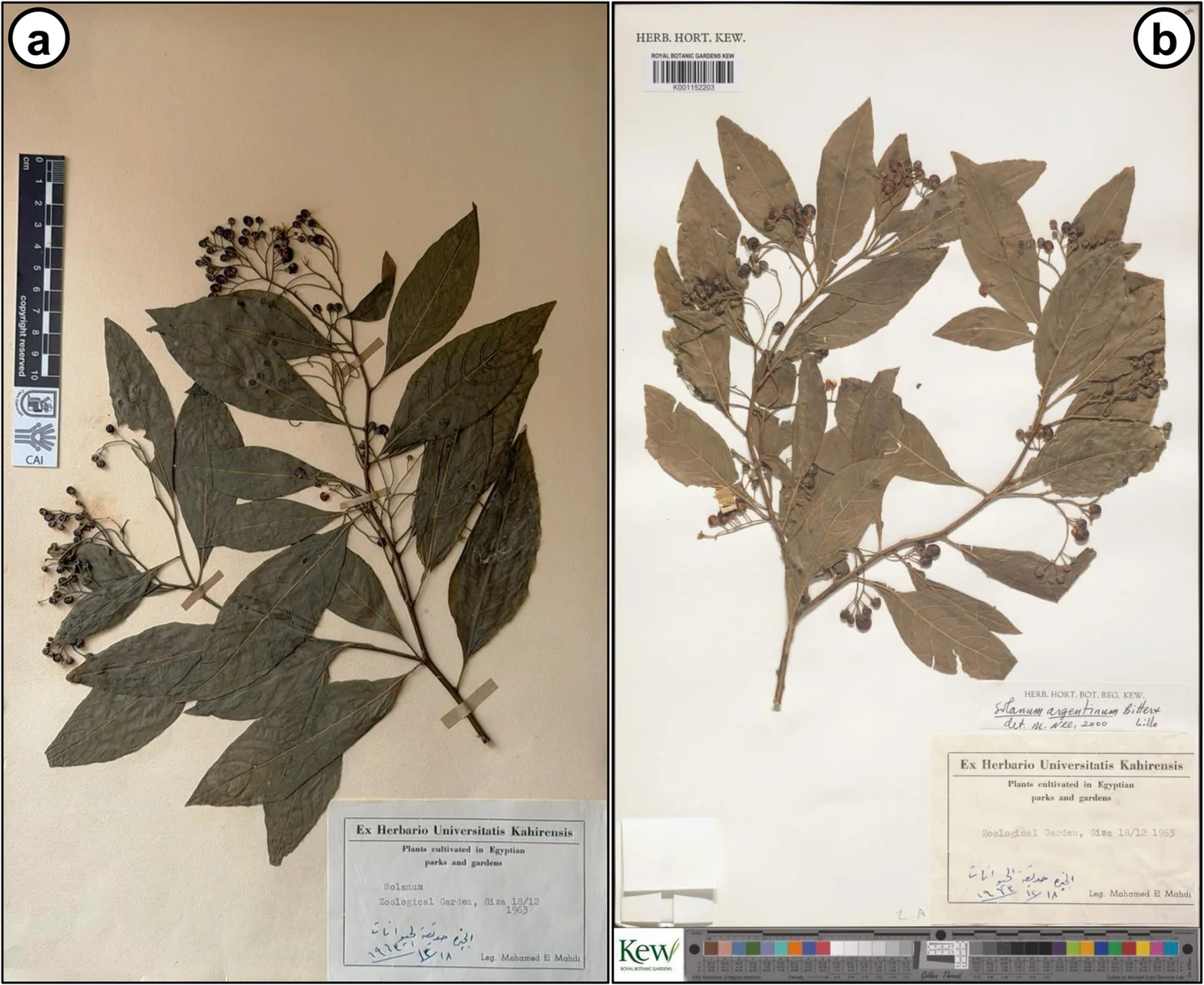
Herbarium specimens of *Solanum argentinum*. **a**, deposited at CAI; **b**, deposited at Kew with accession (K001152203). The specimen was collected by Mohamed El Mahdi s.n. from the Zoological Garden, Giza zoo, Egypt (December 18, 1963) and was identified at the genus level (as *Solanum* sp.). Later in 2000, the specimen was re-identified by M. Nil as *S. argentinum*

The occurrence of *S. argentinum* in the Giza Zoo does not represent the first record of this South American species in Egypt. The re-examination of herbarium specimens at the Royal Botanic Gardens, Kew, and in the CAI herbarium at Cairo University uncovered a specimen collected from the same place (Zoological Garden, Giza Zoo, Egypt) on December 18, 1963, by Mohamed El Mahdi *s.n.* (K001152203) [83] and originally identified only to the genus level, *Solanum* sp. (Fig. 9a). This specimen was re-identified later in 2000 by M. Nil as *S. argentinum* (Fig. 9b). This historical record confirms the species’ presence in Egypt for over 60 years, and yet it has remained unrecorded in the published literature. Thus, the current study presents the first formally published record of *S. argentinum* for Egypt, establishing it as a newly introduced alien species.

Zoological gardens typically host many alien ornamental plants, often introduced intentionally through horticultural trade [84–86]. Climate change increases the risk of ornamental plants invasion, which were previously introduced and cultivated by a local botanical garden or nursery and then escaped into the field after acclimating to the new habitat [87, 88]. Alternative dispersal routes may include introduction via contaminated fodder accompanying animals or bedding materials imported from abroad [84, 89, 90]. These introductions were accompanied by successful establishment in disturbed, irrigated, nutrient-enriched habitats that mirror ruderal conditions [91]. A survey of South African national botanical and zoological gardens recorded 225 alien plant species; almost half were introduced as ornamentals, many of which have escaped cultivation, while others were accidental introductions as contaminants, compounded by heavy international visitation and surrounding urban development [85]. However, the mechanism of the species’ introduction into Egypt is still hypothetical, and the precise timing of this introduction requires further investigation.

The results of our study on the vegetative and reproductive parts of the introduced taxon were, in general, consistent with previous descriptions of Bitter [36] and Knapp [6] of *S. argentinum*. The study provided a detailed description of the micro-morphology of the leaves, pollen grains, and seeds. The studied species exhibited diagnostic morphological characteristics that link them to the Geminata group, differentiating them from other *Solanum* groups. These diagnostic morphological characteristics include the plant structure (unarmed woody habit); the indumentum (trichome types and absence of stellate trichomes); the shape and color of the corolla and the fruits; and the shape and number of the seeds.

Our LM and SEM observations showed that pollen grains were tricolporate, prolate, and small, with scabrate ornamentation. Several studies have explored the pollen morphology of the genus *Solanum* [92–97]. However, to our knowledge, no research has investigated the pollen characteristics of *S. argentinum* before. The pollen grains are released by shaking out through vibratile pollination [98, 99]. This type of pollen release was correlated with the small size of the pollen grains and their little or no ornamentation, as reported by Symon [100]. Tiantian, et al. [101] studied the pollen morphology of 67 species of the genus *Solanum* L. and reported that *S. pseudocapsicum* and related group had small, tricolporate, granulate-ornamented pollen grains. Our examination of *S. argentinum* revealed a similar stamen structure, pollen release type, and pollen characteristics, in agreement with previous studies.

The examined fruits contained a small number of seeds (4–8) per berry. Seed number per fruit is a key morphological feature to distinguish between *Solanum* clades. For example, the Geminata clade has relatively few seeds per fruit compared to the Morelloid clade, which contains more than 50 seeds per fruit [102].

Several reports suggested that seed morphology is a useful character for the taxonomy of the genus *Solanum* [103–106], and it can be utilized to delimit morphological groups in some clades, such as the Geminata clade [6, 8]. Knapp [8] described the seeds of the *S. pseudocapsicum* group as flattened-reniform, pale yellowish-brown, with pitted surfaces. Our seed morphology results under LM and SEM are consistent with the seed-coat patterns of other species in the genus *Solanum* [104, 106–108].

The present study reported that both glandular and non-glandular trichomes were observed on *S. argentinum*, such as unicellular, multicellular uniseriate, bicellular furcate, and dendritic non-glandular trichomes, as well as uniseriate with a unicellular head glandular trichomes (Fig. 5). However, previous studies cited only simple and dendritic non-glandular trichomes on the stem and leaves, with trichome length ranging from 0.25 to 1 mm [54]. Our findings reported that shorter trichomes vary from 0.055 mm in unicellular trichomes to 0.207 mm in dendritic trichomes. Trichome types and their distribution also varied among different vegetative and floral organs. Interestingly, trichome distribution differs markedly between the adaxial and abaxial leaf surfaces of *S. argentinum*. Several authors have reported the importance of the indumentum covering the surface of the genus *Solanum*, which provides valuable sources of information for the systematics of the genus *Solanum* [12, 14–18]. The wide diversity of trichome types enabled taxonomists to distinguish different clades within the genus *Solanum*. For example, stellate hairs characterize the members of the Leptostemonum clade, whereas the Geminata clade contains simple and dendritic trichomes but never stellate ones [6, 8, 19]. Aliyu Aliero, et al. [109] reported the presence of short glandular trichomes over the entire leaf surfaces of *S. pseudocapsicum*. In this study, the distribution patterns of both non-glandular and glandular trichomes on leaf surfaces agree with those reported by Solis, et al. [38] and Rigonatto, et al. [37].

The types, distribution, and density of stomata and epidermal cells on both the adaxial and abaxial surfaces are distinctive. The leaves of *S. argentinum* are hypostomatic, which is the most common pattern observed in Solanaceae [65] and in *Solanum* [15, 110–112].

Foliar epidermal characters have proven highly informative for taxonomy within Solanaceae, particularly in *Solanum*. Our study indicated that both adaxial and abaxial leaf surfaces had polygonal epidermal cells with U-undulated cell boundaries and sunken anticlines. Stomata on the abaxial leaf surface are predominantly anisocytic, while the anomocytic type is also present. Previous work has shown the significance of the foliar epidermis in classifying and identifying species in the family Solanaceae and the genus *Solanum*. For example, multiple stomatal patterns were recorded among species, including the stomatal distribution on one or both sides (hypostomatic or amphistomatic) and the occurrence of various stomatal types. Most studies agreed that the anisocytic type was predominant, with occasional admixture of other types (anomocytic, paracytic, diacytic, and rarely staurocytic) [15, 37, 110, 113–116]. Aliyu Aliero, et al. [109] investigated the foliar micromorphology of *S. pseudocapsicum* and showed that the leaves were characterized by anisocytic stomata that were more abundant on the abaxial surfaces. Moreover, Rigonatto, et al. [37] showed that *S. argentinum* is characterized chiefly by anisocytic stomata, with occasional anomocytic ones. The close agreement between these published patterns and our current observations strengthens the taxonomic conclusion that the *Solanum* taxon collected from Egypt corresponds to *S. argentinum*.

The anatomical features of the stem, petiole, and leaf of the examined materials are consistent with the general anatomy of the family Solanaceae [65, 117]. The occurrence of bicollateral vascular bundles and the presence of pith rays between the bundles are characteristic of the family Solanaceae [65] and the genus *Solanum* [66, 67, 111, 112, 118–120]. Annular collenchyma and idioblasts with sand crystals were also previously recorded in several *Solanum* species [66, 67, 111, 118, 119]. Rigonatto, et al. [37] provided a comprehensive anatomical description of the stem, petiole, and leaves of *S. argentinum* and documented several key features, including bicollateral vascular bundles and crystals. Sanghvi, et al. [121] studied the anatomical structure of *S. pseudocapsicum* and showed that the mesophyll was dorsiventral and consisted of palisade tissue and parenchyma. These observations align with prior anatomical descriptions, confirming the taxon’s identity as *S. argentinum*.

To support the identification of the recorded *Solanum* taxon at the species level, sequences from three DNA markers (ITS2, *rbcL*, and *trnL*) were generated, following previous phylogenetic studies for the genus [29, 33]. These sequences underwent an NCBI BLASTN search that returned the highest identity scores with *Solanum* section *Geminata* sequences (Supplementary Table S2). However, final species assignments relied on the phylogenetic inference (ML and BI) using a concatenated matrix of ITS2, *rbcL*, and *trnL*, yielding enhanced interspecific resolution consistent with prior *Solanum* phylogenies [23, 29, 33, 77, 80, 81]. Although the ILD test provided no evidence of conflict among the three markers (*p* = 1.000), we acknowledge its inherent limitations. The congruence among individual-marker topologies, particularly the consistent placement of *S. argentinum* within the *S. pseudocapsicum* species group across all three single-marker trees (Supplementary Figs. S1–S3) provides stronger biological support for concatenation than the ILD test alone. The resulting ML tree demonstrated strong support for species-level relationships within the *Geminata* clade, with the studied specimens forming a fully supported clade (100% ML/1.0 BI) together with *S. argentinum* GenBank accessions (Fig. 8). The *Geminata* clade was recovered with moderate support (98% ML/1.0 BI) as sister to *S. pseudocapsicum*, consistent with placement in the *S. pseudocapsicum* species group. These phylogenetic results, together with morphological characters and comparisons with taxonomic revisions, support identifying the specimens as *S. argentinum*.

## Conclusion

This study presents the first published floristic record of the South American native *S. argentinum* in Egypt. The identity of this alien species was supported through an integrative taxonomic approaches, including morphological characters (foliar and floral morphology, pollen grains, and seeds), anatomical characters (stem, leaf, and petiole), and multi-locus phylogenetic analysis using ITS2, *rbcL*, and *trnL* molecular markers. Re-examining of the historical herbarium material (Fig. 9) confirms that the species has persisted in the Giza Zoological Garden for over 60 years, despite never being recorded in the Egyptian botanical literature. Knowing that the Egyptian population is small, localized entirely within the Giza Zoo, and dependent on managed irrigation and gardener maintenance, we designate *S. argentinum* as an introduced casual alien. The detailed characterization used in this study provides essential diagnostic tools for future identification, monitoring, and management of this introduced species. Additionally, the study highlights the importance of further floristic surveys and the utility of DNA barcoding for resolving the identity of taxonomically complex species within the genus *Solanum*.

## Future directions

To manage this newly recorded alien species responsibly, we outline several key guidelines for future research: (1) Establishment of continuous field monitoring of the Giza Zoological Garden cohort to track potential population changes and detect any sign of reproductive escape into adjacent urban or natural Egyptian habitats. (2) Initiation of wider floristic and botanical surveys across Egypt to determine if other unrecognized populations of *S. argentinum* exist in cultivation or in the wild. (3) Investigation of human-mediated dispersal pathways, such as international horticultural trade, contaminated animal bedding, or seed impurities, to understand how the species arrived in the Zoo. (4) Performing detailed comparative fruit and seed anatomical studies and a full molecular characterization across all native and introduced *Solanum* species in Egypt to strengthen the regional diagnostic and early detection toolkit.

##  Supplementary Information

Supplementary Material 1. Supplementary Table S1. List of examined specimens of S. argentinum and their localities and voucher data.

Supplementary Material 2. Supplementary Table S2. Complete BLASTN search results for three sequenced markers (ITS2, trnL, and rbcL) of Solanum argentinum generated in this study (accessions PZ273763, PZ302159, PZ302158), listing percentage identity, query coverage, E-value, and closest matching GenBank accessions.

Supplementary Material 3. Supplementary Table S3. Metrics of the analyzed regions used in this study as calculated in Geneious 9.1.2 (length), PAUP* (parsimony analysis statistics), and MEGA 12 (Pairwise divergence using the Bootstrap method).

Supplementary Material 4. Supplementary Figure S1. Maximum Likelihood (ML) phylogeny of Solanum section Geminata and outgroups inferred from the ITS2 dataset (212 bp, 25 taxa). The tree was reconstructed under the Tamura (1992) + G + I model, using MEGA12. Bootstrap support values (1,000 replicates) are shown at nodes. Evolutionary analyses were conducted in MEGA12.

Supplementary Material 5. Supplementary Figure S2. Maximum Likelihood (ML) phylogeny of Solanum section Geminata and outgroups inferred from the trnL dataset (547 bp, 25 taxa). We reconstructed the tree under the Tamura (1992) model with invariable sites (I). Bootstrap support values (1,000 replicates) are shown at nodes. Evolutionary analyses were conducted in MEGA12.

Supplementary Material 6. Supplementary Figure S3. Maximum Likelihood (ML) phylogeny of Solanum section Geminata and outgroups inferred from the rbcL dataset (582 bp, 14 taxa). The tree was reconstructed under the GTR+ I model in MEGA12. Bootstrap support values (1,000 replicates) are shown at nodes. Evolutionary analyses were conducted in MEGA12.

Supplementary Material 7. Supplementary material S1.

Supplementary Material 8. Supplementary material S2.

## Data Availability

The datasets produced and/or analyzed during this study are available from the corresponding author upon reasonable request.

## Abbreviations

BI: Bayesian inference
ITS2: Internal transcribed spacer
LM: Light microscopy
ML: Maximum likelihood
PCR: Polymerase chain reaction
SEM: Scanning electron microscopy
