# Supplementary material for: Morpho-anatomical characterization, DNA barcoding, and phylogenetic analysis of Solanum argentinum Bitter & Lillo: an addition to the alien Flora of Egypt
Source: BMC Plant Biol. 2026 Nov 3;26:1882. doi: 10.1186/s12870-026-10047-1 (PMC13733171; doi:10.1186/s12870-026-10047-1)
Supplement: Supplementary file 1 [file 12870_2026_10047_MOESM4_ESM.pdf]

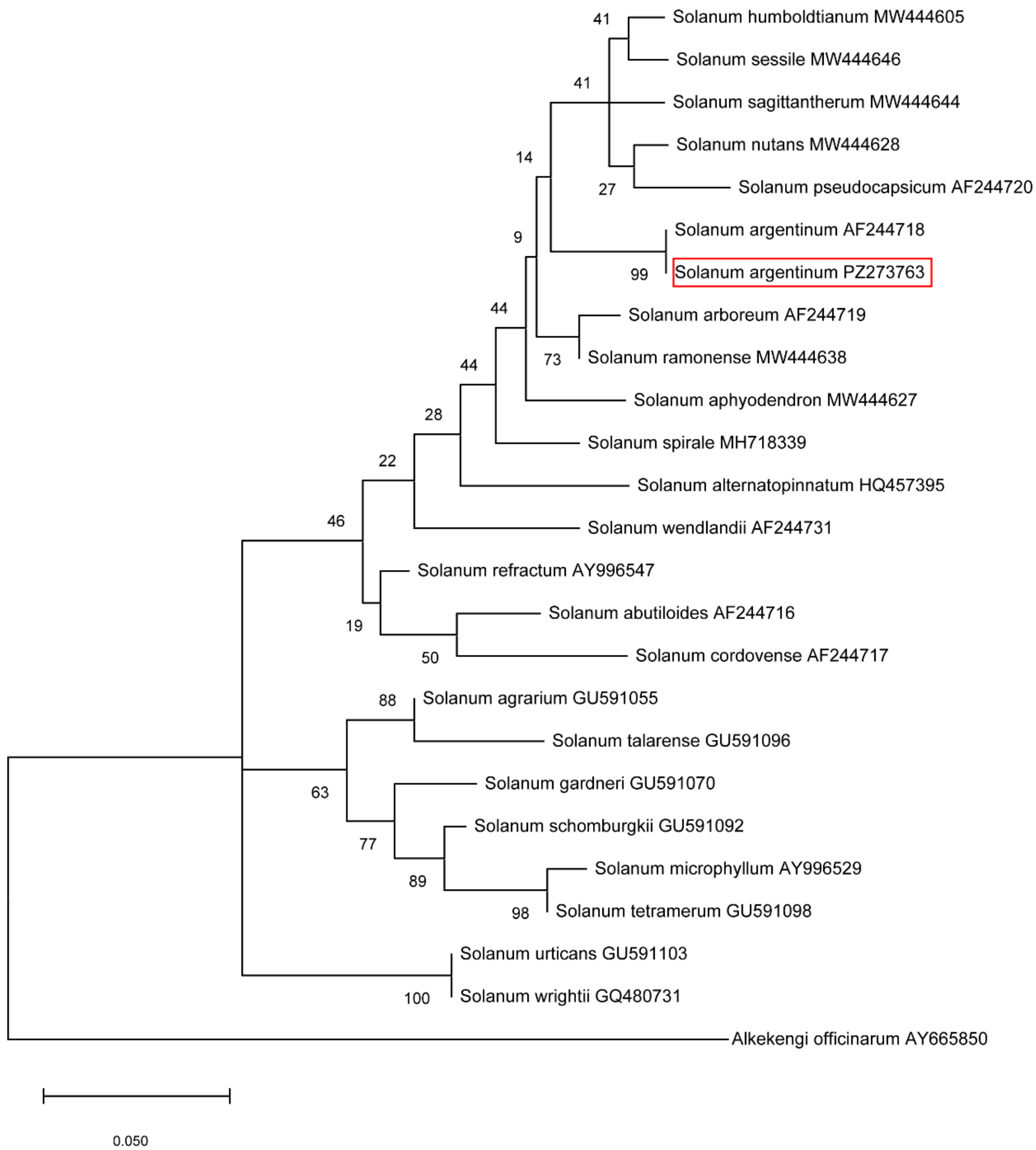

Supplementary Figure S1. Maximum Likelihood (ML) phylogeny of *Solanum* section *Geminata* and outgroups inferred from the ITS2 dataset (212 bp, 25 taxa). The tree was reconstructed under the Tamura (1992) + G + I model, in MEGA12. Bootstrap support values (1,000 replicates) are shown at nodes. Evolutionary analyses were conducted in MEGA12.
