# Supplementary material for: Morpho-anatomical characterization, DNA barcoding, and phylogenetic analysis of Solanum argentinum Bitter & Lillo: an addition to the alien Flora of Egypt
Source: BMC Plant Biol. 2026 Nov 3;26:1882. doi: 10.1186/s12870-026-10047-1 (PMC13733171; doi:10.1186/s12870-026-10047-1)
Supplement: Supplementary file 2 [file 12870_2026_10047_MOESM5_ESM.pdf]

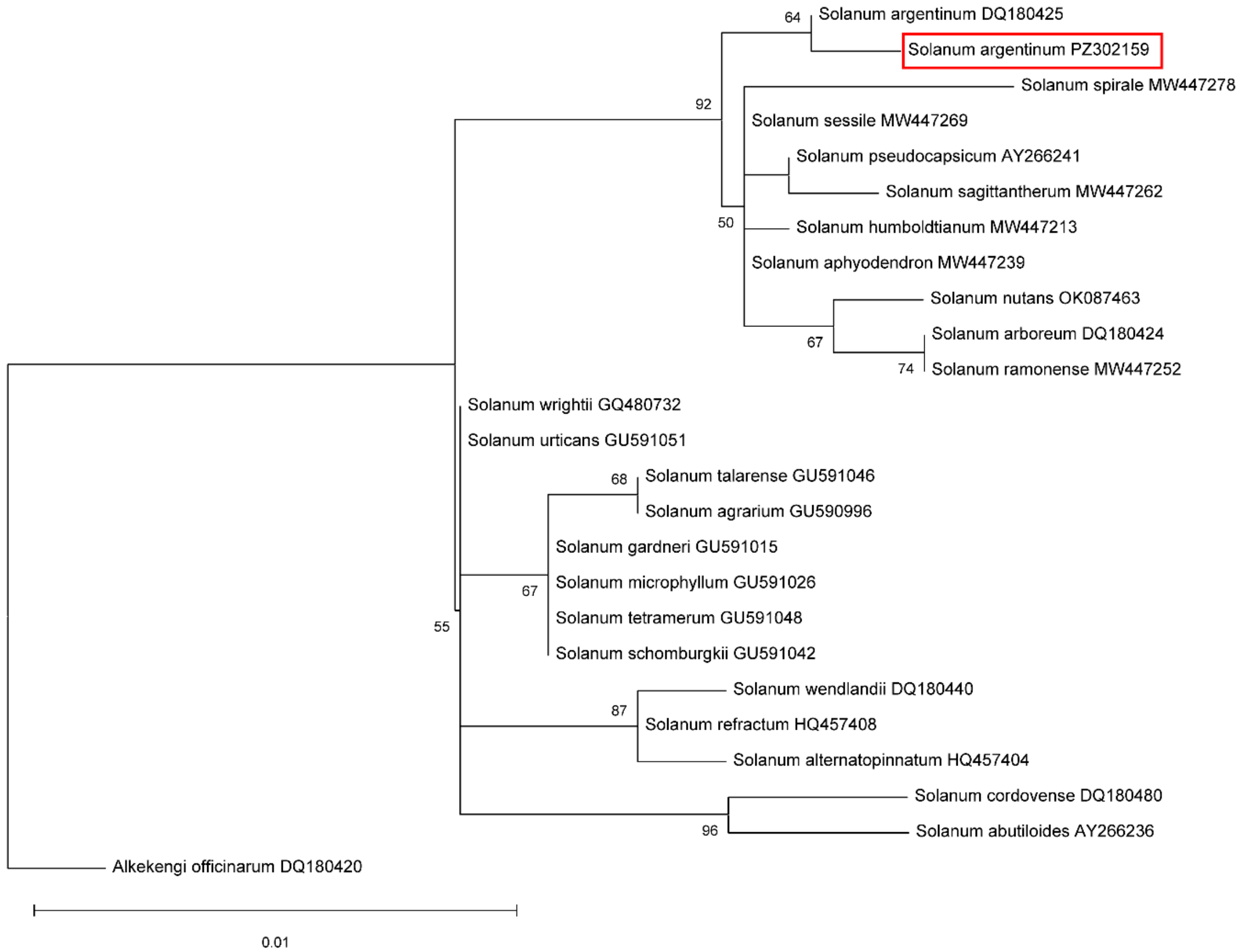

Supplementary Figure S2. Maximum Likelihood (ML) phylogeny of *Solanum* section *Geminata* and outgroups inferred from the *trnL* dataset (547 bp, 25 taxa). We reconstructed the tree under the Tamura (1992) model with invariable sites (I). Bootstrap support values (1,000 replicates) are shown at nodes. Evolutionary analyses were conducted in MEGA12.
