# Supplementary material for: Morpho-anatomical characterization, DNA barcoding, and phylogenetic analysis of Solanum argentinum Bitter & Lillo: an addition to the alien Flora of Egypt
Source: BMC Plant Biol. 2026 Nov 3;26:1882. doi: 10.1186/s12870-026-10047-1 (PMC13733171; doi:10.1186/s12870-026-10047-1)
Supplement: Supplementary file 4 [file 12870_2026_10047_MOESM1_ESM.docx]

Supplementary Table S2. Complete BLASTN search results for three sequenced markers (ITS2, *trnL*, and *rbcL*) of *Solanum argentinum* generated in this study (accessions PZ273763, PZ302159, PZ302158), listing percentage identity, query coverage, E‑value, and closest matching GenBank accessions.

| **Sequence ID** | **Marker** | **GenBank closest match (accession)** | **Query coverage (%)** | **E‑value** | **% Identity** | **Accession length** |
| --- | --- | --- | --- | --- | --- | --- |
| *Solanum argentinum* Bitter & Lillo [PZ273763] | ITS2 | *Solanum argentinum* Bitter & Lillo [AF244718] | 97% | 8.00E-94 | 100.00% | 616 |
|  |  | *Solanum alternatopinnatum* Steud [HQ457395] | 100% | 1.00E-63 | 92.27% | 640 |
|  |  | *Solanum agrarium* Sendtn. [GU591055] | 100% | 2.00E-105 | 91.67% | 631 |
|  |  | *Solanum aphyodendron* S.Knapp [MW444627] | 100% | 3.00E-75 | 96.11% | 642 |
|  |  | *Solanum arboreum* Humb. & Bonpl. ex Dunal [AF244719] | 96% | 1.00E-74 | 97.11% | 617 |
|  |  | *Solanum argentinum* Bitter & Lillo [AF244718] | 96% | 4.00E-83 | 100.00% | 616 |
|  |  | *Solanum cordovense* Sessé & Moc. [AF244717] | 97% | 5.00E-72 | 89% | 611 |
|  |  | *Solanum gardneri* Sendtn. [GU591070] | 100% | 6.00E-62 | 88.89% | 635 |
|  |  | *Solanum humboldtianum* Granados-Tochoy & S.Knapp [MW444605] | 100% | 1.00E-73 | 95.56% | 637 |
|  |  | *Solanum microphyllum* Dunal [AY996529] | 100% | 3.00E-55 | 86.67% | 632 |
|  |  | *Solanum nutans* Ruiz & Pav. [MW444628] | 100% | 1.00E-73 | 95.56% | 640 |
|  |  | *Solanum pseudocapsicum* L. [AF244720] | 96% | 4.00E-64 | 93.64% | 613 |
|  |  | *Solanum ramonense* C.V.Morton & Standl. [MW444638] | 100% | 2.00E-77 | 96.67% | 641 |
|  |  | *Solanum refractum* Hook. & Arn. [AY996547] | 100% | 6.00E-67 | 93.33% | 634 |
|  |  | *Solanum sagittantherum* Granados-Tochoy & C.I.Orozco [MW444644] | 100% | 4.00E-64 | 92.31% | 635 |
|  |  | *Solanum schomburgkii* Sendtn. [GU591092] | 100% | 3.00E-60 | 88.33% | 632 |
|  |  | *Solanum sessile* Ruiz & Pav. [MW444646] | 100% | 8.00E-71 | 94.44% | 634 |
|  |  | *Solanum spirale* Roxb. [MH718339] | 100% | 3.00E-70 | 94.44% | 437 |
|  |  | *Solanum talarense* Svenson [GU591096] | 100% | 2.00E-60 | 88.89% | 631 |
|  |  | *Solanum tetramerum* Dunal [GU591098] | 100% | 8.00E-56 | 86.67% | 625 |
|  |  | *Solanum urticans* Dunal [GU591103] | 100% | 1.00E-59 | 88.40% | 637 |
|  |  | *Solanum wendlandii* Hook.f. [AF244731] | 96% | 5.00E-66 | 91.38% | 613 |
|  |  | *Solanum wrightii* Benth. [GQ480731] | 100% | 2.00E-57 | 88.40% | 637 |
|  |  | *Alkekengi officinarum* Moench [AY665850] | 100% | 3.00E-46 | 84.53% | 644 |
| *Solanum argentinum* Bitter & Lillo [PZ302159] | *trnL* | *Solanum argentinum* Bitter & Lillo [DQ180425] | 100% | 0.00E+00 | 99.82% | 1709 |
|  |  | *Solanum abutiloides* Bitter & Lillo [AY266236] | 100% | 0 | 98.72% | 1711 |
|  |  | *Solanum agrarium Sendtn.* [GU590996] | 99% | 0.00E+00 | 97.97% | 1708 |
|  |  | *Solanum alternatopinnatum* Steud. [HQ457404] | 100% | 0.00E+00 | 98.35% | 1621 |
|  |  | *Solanum aphyodendron* S.Knapp [MW447239] | 100% | 0.00E+00 | 99.08% | 1621 |
|  |  | *Solanum arboreum* Humb. & Bonpl. ex Dunal [DQ180424] | 100% | 0.00E+00 | 99.08% | 1753 |
|  |  | *Solanum cordovense* Sessé & Moc. [DQ180480] | 100% | 0.00E+00 | 97.99% | 1762 |
|  |  | *Solanum gardneri* Sendtn. [GU591015] | 100% | 0.00E+00 | 98.72% | 1711 |
|  |  | *Solanum humboldtianum* Granados-Tochoy & S.Knapp [MW447213] | 100% | 0.00E+00 | 99.27% | 1621 |
|  |  | *Solanum microphyllum* Dunal [GU591026] | 100% | 0.00E+00 | 98.72% | 1711 |
|  |  | *Solanum nutans* Ruiz & Pav. [OK087463] | 100% | 0.00E+00 | 99.08% | 1589 |
|  |  | *Solanum pseudocapsicum* L. [AY266241] | 100% | 0.00E+00 | 99.45% | 1725 |
|  |  | *Solanum ramonense* C.V.Morton & Standl. [MW447252] | 100% | 0.00E+00 | 98.90% | 1625 |
|  |  | *Solanum refractum* Hook. & Arn. [HQ457408] | 100% | 0.00E+00 | 98.53% | 1609 |
|  |  | *Solanum sagittantherum* Granados-Tochoy & C.I.Orozco [MW447262] | 100% | 0.00E+00 | 99.08% | 1621 |
|  |  | *Solanum schomburgkii* Sendtn. [GU591042] | 100% | 0.00E+00 | 98.72% | 1711 |
|  |  | *Solanum sessile* Ruiz & Pav. [MW447269] | 100% | 0.00E+00 | 99.27% | 1621 |
|  |  | *Solanum spirale* Roxb. [MW447278] | 100% | 0.00E+00 | 98.90% | 1621 |
|  |  | *Solanum talarense* Svenson [GU591046] | 99% | 0.00E+00 | 97.97% | 1613 |
|  |  | *Solanum tetramerum* Dunal [GU591048] | 100% | 0.00E+00 | 98.72% | 1711 |
|  |  | *Solanum urticans* Dunal [GU591051] | 100% | 0.00E+00 | 98.90% | 1685 |
|  |  | *Solanum wendlandii* Hook.f. [DQ180440] | 100% | 0.00E+00 | 98.35% | 1757 |
|  |  | *Solanum wrightii* Benth. [GQ480732] | 100% | 0.00E+00 | 98.90% | 1833 |
|  |  | *Alkekengi officinarum* Moench [DQ180420] | 100% | 0.00E+00 | 98.17% | 1820 |
| *Solanum argentinum* Bitter & Lillo [PZ302158] | *rbcL* | *Solanum aphyodendron* S.Knapp [OP710555] | 94% | 0.00E+00 | 98.71% | 547 |
|  |  | *Solanum cordovense* Sessé & Moc. [JQ594150] | 91% | 0.00E+00 | 98.10% | 552 |
|  |  | *Solanum pseudocapsicum* L. [HM850368] | 100% | 0.00E+00 | 99.13% | 1363 |
|  |  | *Solanum ramonense* C.V.Morton & Standl. [JQ594556] | 91% | 0.00E+00 | 99.62% | 552 |
|  |  | *Solanum spirale* Roxb. [JF944462] | 100% | 0.00E+00 | 99.48% | 615 |
|  |  | *Solanum talarense* Svenson [MZ221863] | 100% | 0.00E+00 | 98.62% | 155289 |
|  |  | *Solanum wrightii* Benth. [MH722386] | 100% | 0.00E+00 | 98.79% | 698 |
|  |  | *Alkekengi officinarum* Moench [U08617] | 100% | 0.00E+00 | 97.40% | 1408 |
