# Supplementary material for: Morpho-anatomical characterization, DNA barcoding, and phylogenetic analysis of Solanum argentinum Bitter & Lillo: an addition to the alien Flora of Egypt
Source: BMC Plant Biol. 2026 Nov 3;26:1882. doi: 10.1186/s12870-026-10047-1 (PMC13733171; doi:10.1186/s12870-026-10047-1)
Supplement: Supplementary file 5 [file 12870_2026_10047_MOESM2_ESM.docx]

**Supplementary Table S1.** List of examined specimens of *S. argentinum*, their localities, and voucher data.

| **Localities** | **Collection date** | **Collector name** | **Voucher number** | **Herbarium acronym** |
| --- | --- | --- | --- | --- |
| Zoological Garden (Giza Zoo), Giza, Egypt  30°01'40.2"N, 31°12'54.6"E | 17.08.1963 | M. El Mahdi | s.n. | CAI |
|  | 18.12.1963 | M. El Mahdi | s.n. | CAI |
|  | 25.06.1967 | M. Ezz Eldin | s.n. | Orman Herbarium |
|  | 02.06.1974 | B. Diwan | s.n. | Orman Herbarium |
|  | 08.02.2007 | Herbarium groups | s.n. | Orman Herbarium |
|  | 16.03.2008 | Herbarium groups | s.n. | Orman Herbarium |
|  | 05.04.2022 | Hamdy, R.S. | s.n. | CAI |
|  | 05.04.2022 | Hamdy, R.S. | ASTU-MA1001* | ASTU |
|  | 02.07.2023 | Hamdy, R.S. | s.n. | CAI |
|  | 02.07.2023 | Hamdy, R.S. | QNA-S113 | QNA |
|  | 17.08.2023 | Hamdy, R.S. | s.n. | CAI |
|  | 17.08.2023 | Hamdy, R.S. | QNA-S114 | QNA |
|  | 07.06.2024 | Hamdy, R.S. | s.n. | CAI |
|  | 07.06.2024 | Hamdy, R.S. | ASTU-MA1002 | ASTU |

*The specimen deposited in the ASTU herbarium was used for sequence analysis.
