# Supplementary material for: Morpho-anatomical characterization, DNA barcoding, and phylogenetic analysis of Solanum argentinum Bitter & Lillo: an addition to the alien Flora of Egypt
Source: BMC Plant Biol. 2026 Nov 3;26:1882. doi: 10.1186/s12870-026-10047-1 (PMC13733171; doi:10.1186/s12870-026-10047-1)
Supplement: Supplementary file 6 [file 12870_2026_10047_MOESM3_ESM.docx]

Supplementary Table S3. Metrics of the analyzed regions used in this study as calculated in Geneious 9.1.2 (length), PAUP* (parsimony analyses statistics), and MEGA 12 (Pairwise divergence using the Bootstrap method).

| **Region** | **Aligned sequence length** | **Pairwise divergence** | **Constant characters** | **CI** | **HI** | **CI excluding uninformative characters.** | **HI, excluding uninformative characters** | **RI** | **RC** |
| --- | --- | --- | --- | --- | --- | --- | --- | --- | --- |
| **ITS2** | 212 | 0.098 | 145 | 0.5833 | 0.4167 | 0.4922 | 0.5078 | 0.6919 | 0.4036 |
| ***trnL*** | 547 | 0.0097 | 515 | 1.0000 | 0.0000 | 1.0000 | 0.0000 | 1.0000 | 1.0000 |
| ***rbcL*** | 582 | 0.013 | 559 | 0.9630 | 0.0370 | 0.9167 | 0.0833 | 0.9286 | 0.8942 |
| **combined** | 1290 | 0.031 | 1169 | 0.6742 | 0.3258 | 0.5528 | 0.4472 | 0.7363 | 0.4964 |

Abbreviations: CI=Consistency index; HI=Homoplasy index, RI= Retention Index, RC=Rescaled consistency index.
